# Supplementary material for: Seafloor evidence for pre-shield volcanism above the Tristan da Cunha mantle plume
Source: Nat Commun. 2020 Sep 11;11:4543. doi: 10.1038/s41467-020-18361-4 (PMC7486381; doi:10.1038/s41467-020-18361-4)
Supplement: Supplementary file 1 — Supplementary Information [file 41467_2020_18361_MOESM1_ESM.pdf]

Supplementary Information for

Seafloor evidence for pre-shield volcanism above the Tristan da Cunha mantle plume

by Geissler et al.

The supplementary material presents figures, tables, notes and references that complement the main manuscript.

Supplementary Figure 1 illustrates seamounts in the Tristan da Cunha area. Supplementary Figure 2 presents seabed backscatter for the complete study area. Supplementary Figure 3 shows unclassified backscatter mosaics of all study areas. Supplementary Figure 4 presents evidence for past slope instability around Tristan da Cunha. Supplementary Figure 5 compares the water depths from our bathymetry data with a global digital elevation model. Supplementary Figure 6 shows the distribution of seabed backscatter. Supplementary Note 1 and Supplementary Figure 7 introduce and discuss aspects of combining seabed backscatter data from different cruises and slightly different sensors in more detail.

All supplementary figures in high resolution, and the digital terrain model, as well as the backscatter mosaic are retrievable from the PANGAEA archive as mentioned in the chapter data availability[1].

Supplementary Table S1 contains the volume calculations of the distinguished volcanic cones while Supplementary Table 2 displays the decision table to calculate the benthic terrain model.

The supplementary references complete the given information.

Supplementary Figures

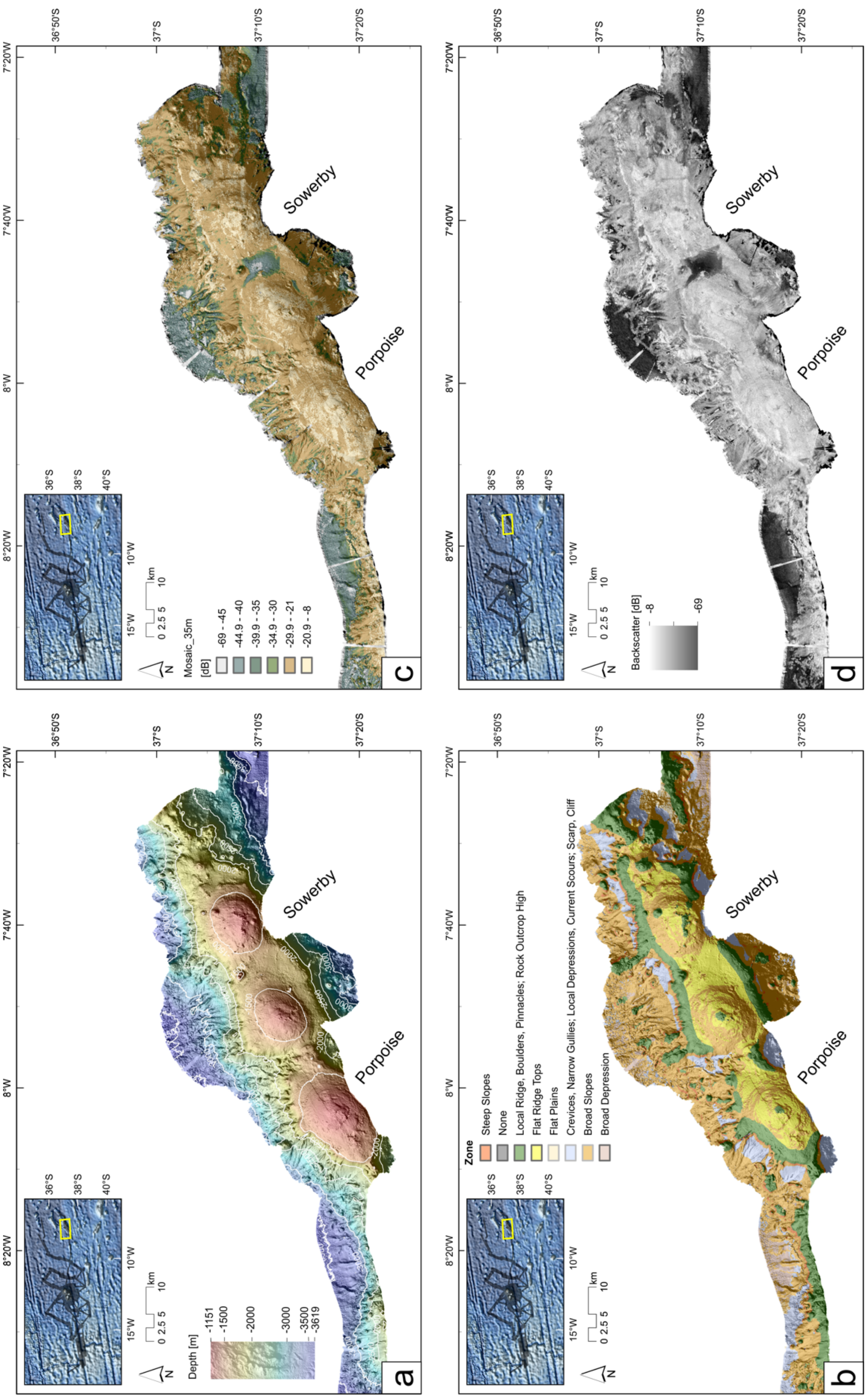

Supplementary Figure 1 | Seamounts to the east of Tristan da Cunha. (a) High-resolution bathymetry for seamounts east of Tristan da Cunha. (**b**) Benthic Terrain Model. (**c**) Backscatter intensity classified. Brownish colors show areas with high backscatter amplitudes. (**d**) Backscatter in greyscale (linear) unclassified.

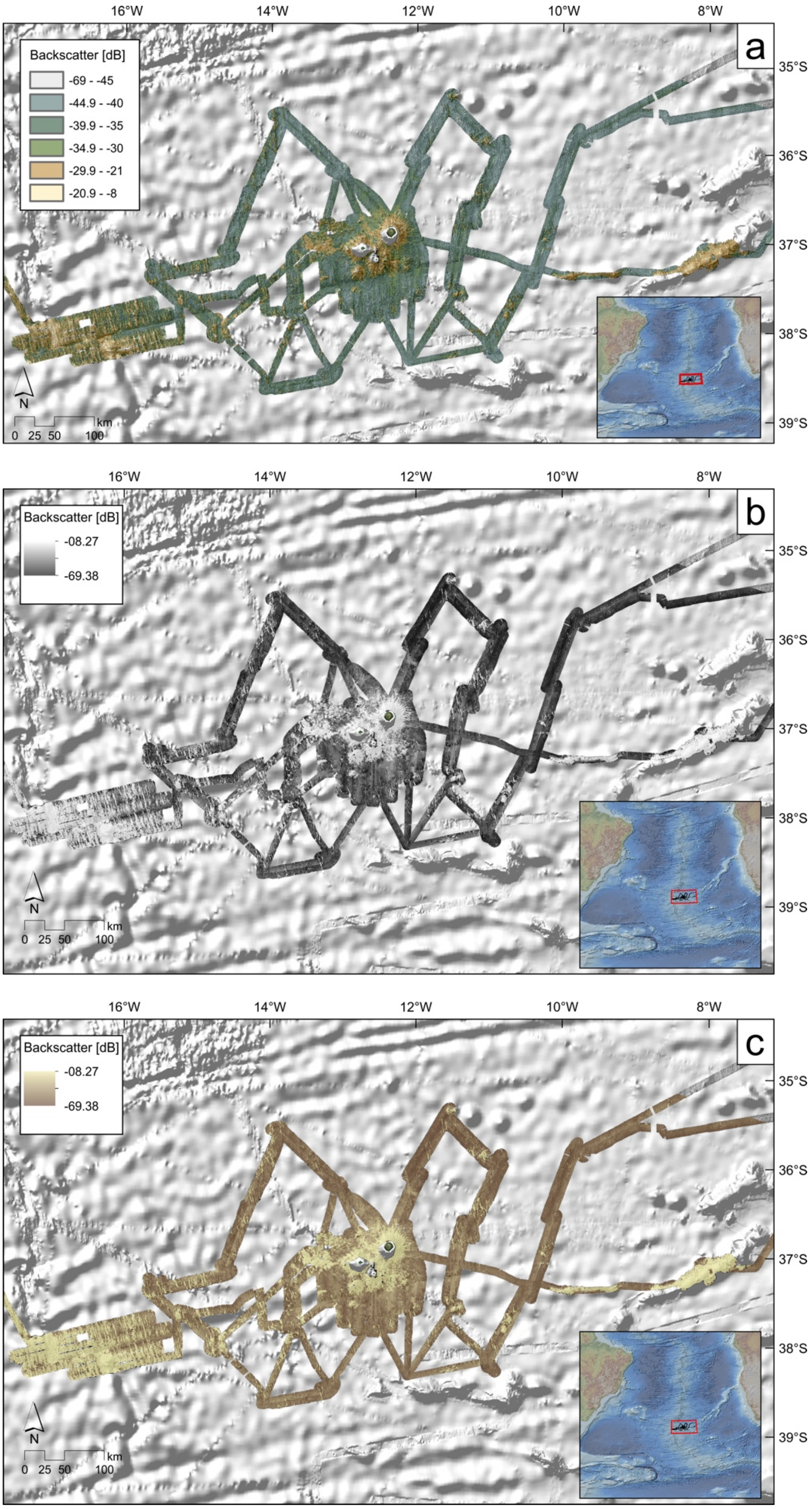

Supplementary Figure 2 | Backscatter mosaic overview. Backscatter mosaic overview in (**a**) colored, classified, (**b**) linear greyscale unclassified, and (**c**) linear white-to-brown-scale unclassified.

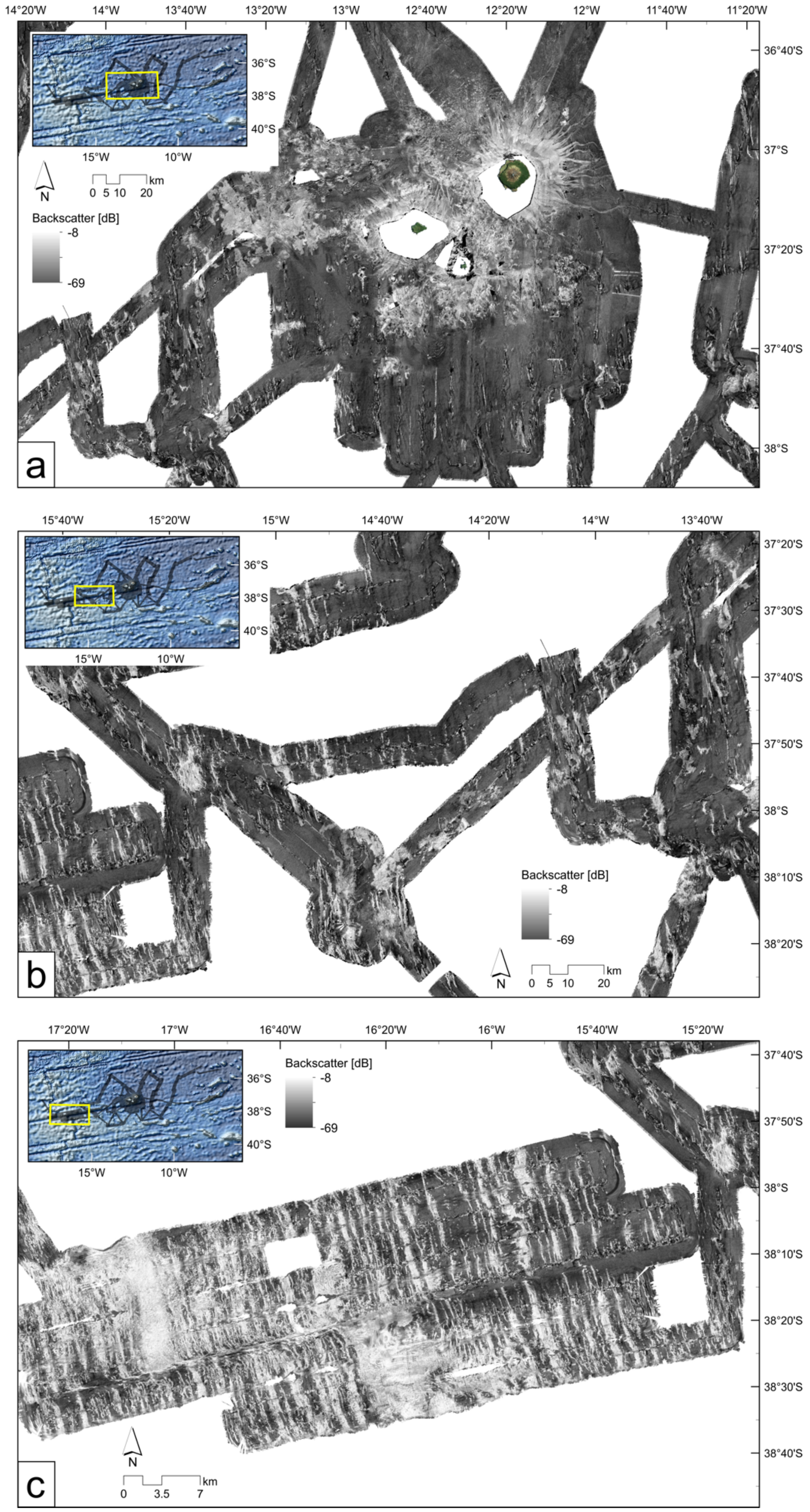

Supplementary Figure 3 | Unclassified backscatter. Unclassified backscatter of (**a**) the central region around Tristan da Cunha; (**b**) the central west and, (**c**) the area around the Mid-Atlantic Ridge and the core complex. The greyscale is linear.

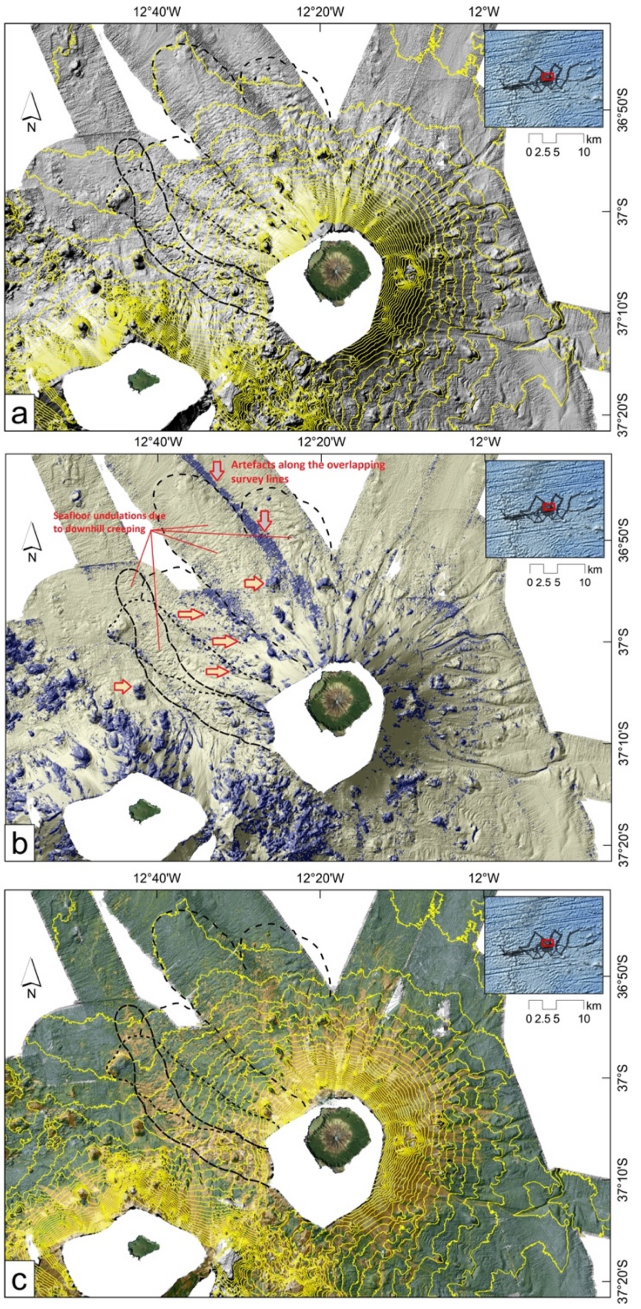

Supplementary Figure 4 | GIS based analysis of the NW flank of Tristan da Cunha. The northern submarine flank of Tristan da Cunha seems to have been affected by numerous mass transport events. It shows several scarps, blocks and lobes that reveal the occurrence of downslope transport by sliding and creep-like processes. The variable backscatter intensity in this area supports the presence of variable thicknesses of sediment drape that imply mass transport occurred in several phases over a long period of time.

(**a**) Hillshade and depth contour lines around Tristan da Cunha. (**b**) Drape of the most rugged areas, characterized by volcanic outcrop and the presence of variably-sized boulders (in blue and partly indicated by horizontal arrows) based on the analysis of Surface Area to Planar Area[2], which computes a ratio between the three-dimensional surface area and the planar area of the surface. (**c**) Depth contour lines overlain on seabed backscatter intensity. The dashed and solid black lines in all three figures outline different debris avalanche deposits.

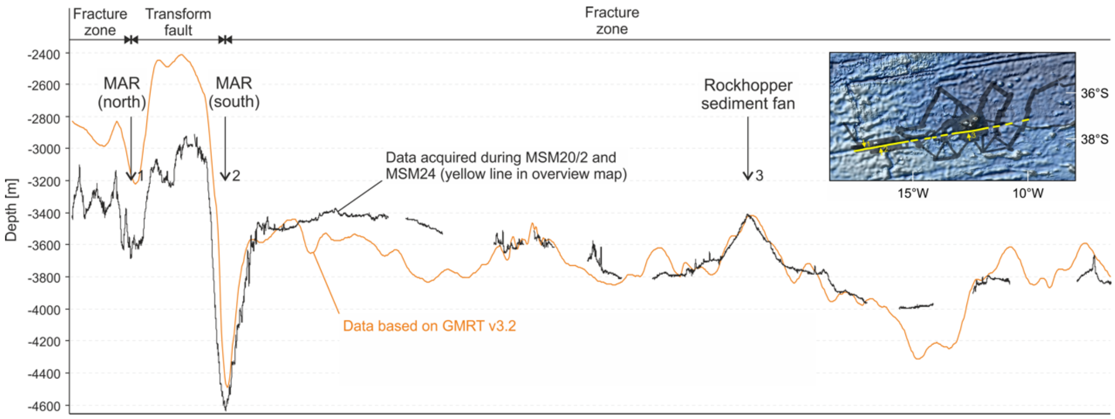

Supplementary Figure 5 | Water depth along the Tristan da Cunha Transform Fault and Fracture Zone System. This figure compares the global Digital Elevation Model GMRT[3] and our measured bathymetry along the Tristan da Cunha Transform Fault and Fracture Zone System (TTFZ). The GMRT[3] water depths in the region around Tristan da Cunha are mainly derived from satellite altimetry due to the lack of ship-based bathymetry. Smith and Sandwell[4] stated that they excluded specific areas (i.e. Walvis Ridge) from their determination of bathymetry by inversion of satellite-derived free-air gravity data due to anomalous crustal thicknesses. It therefore seems very likely that some smaller areas of anomalous crustal thickness, such as those in the region of the Tristan da Cunha hot spot, have been treated in insufficient detail for the focus of a regional study such as ours.

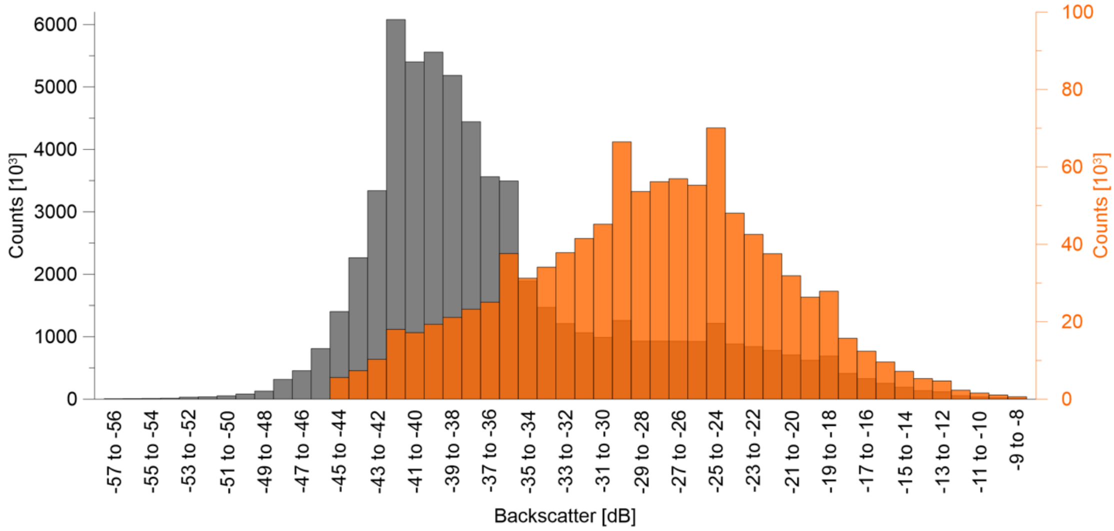

Supplementary Figure 6 | Seabed backscatter histogram. Seabed backscatter values of the whole study area (grey color) compared to backscatter values of the mapped volcanic cones (orange). Latter show a higher mean backscatter.

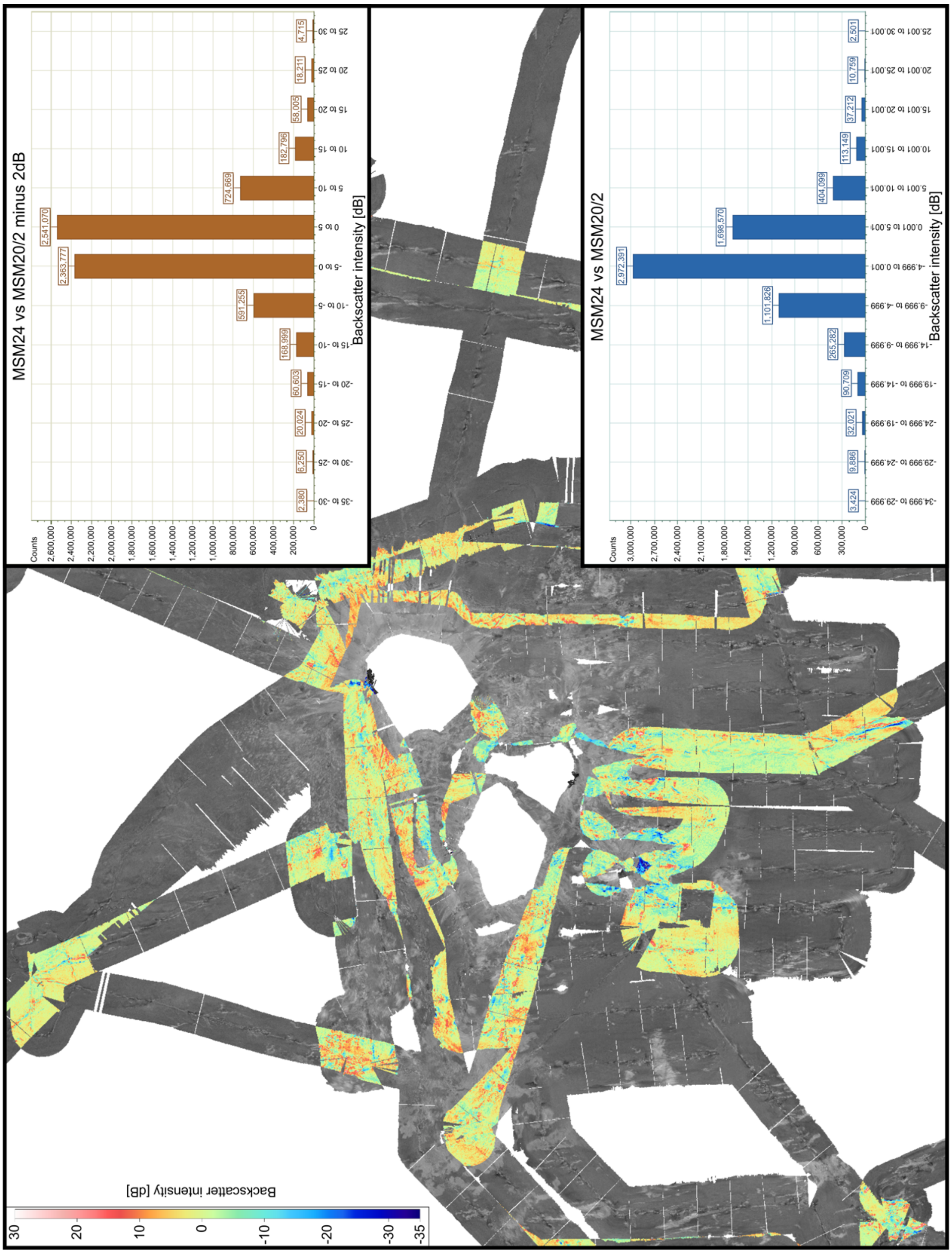

Supplementary Figure 7 | Combined backscatter mosaic. Comparison of seabed backscatter of cruises MSM24 and MSM20/2. Overlapping survey areas are colored according to the dB values of the offset-corrected histogram in the upper right corner. The uncorrected histogram is shown in the lower right corner.

Supplementary Tables

Supplementary Table 1 | Volume calculations of volcanic cones. Geographical position, backscatter value and describing geomorphological parameters of all mapped volcanic cones. Isolde Smt. (#262) and Rockhopper Smt. (#279) are highlighted in grey.

Supplementary Table 2 | Decision table. Decision table summarizing the factors used in the structural Benthic Terrain Model classification (modified after Erdey-Heydorn[5]). The Bathymetric Positioning Index values are given in grid units (standardized value of each data point was multiplied by 100) and represent ±1 standard deviation. Slope values are given in degrees. For a better visualization, some of the structural zones are grouped together in the final map.

Supplementary Table 1: Volume calculations of volcanic cones. Isolde Smt. (#262) and Rockhopper Smt. (#279) are highlighted in grey.

#	Lat [DDD]	Lon [DDD]	Total volume [km³]	**Net volume [km³]**	Cut volume [km³]	Cut area [km²]	Cut area 3D [km²]	Fill volume [km³]	Fill area [km²]	Fill area 3D [km²]	**Enclosed area [km²]**	Peri-meter [km]	**Mean diameter [km]**	Back-scatter [dB]
1	-37.2413	-12.3974	0.010	**0.010**	0.010	0.275	0.297	0.00024	0.064	0.067	**0.340**	2.106	**0.670**	-30.06
2	-37.5981	-13.0440	0.006	**0.005**	0.006	0.272	0.284	0.00049	0.068	0.070	**0.340**	2.106	**0.670**	-37.04
3	-37.1545	-12.9391	0.012	**0.011**	0.011	0.331	0.357	0.00012	0.048	0.049	**0.380**	2.208	**0.703**	-35.46
4	-37.0146	-12.4787	0.038	**0.037**	0.037	0.636	0.704	0.00007	0.036	0.037	**0.673**	2.943	**0.937**	-28.60
5	-37.1472	-13.0845	0.028	**0.028**	0.028	0.547	0.598	0.00015	0.027	0.029	**0.576**	2.751	**0.876**	-27.80
6	-37.1871	-13.1362	0.031	**0.031**	0.031	0.570	0.635	0.00012	0.056	0.057	**0.626**	2.842	**0.905**	-37.41
7	-37.2112	-13.2575	0.021	**0.020**	0.020	0.455	0.489	0.00031	0.093	0.095	**0.548**	2.662	**0.847**	-29.15
8	-37.2085	-13.2675	0.035	**0.035**	0.035	0.622	0.692	0.00032	0.070	0.072	**0.693**	2.981	**0.949**	-31.81
9	-36.7823	-12.4374	0.017	**0.016**	0.017	0.475	0.514	0.00048	0.121	0.122	**0.597**	2.772	**0.882**	-30.88
10	-36.7994	-12.4653	0.016	**0.015**	0.016	0.525	0.549	0.00029	0.147	0.148	**0.673**	2.951	**0.939**	-27.23
11	-36.9862	-12.4962	0.024	**0.023**	0.024	0.509	0.576	0.00034	0.081	0.084	**0.591**	2.744	**0.873**	-27.15
12	-37.0515	-12.5214	0.027	**0.025**	0.026	0.567	0.626	0.00071	0.194	0.199	**0.762**	3.205	**1.020**	-32.78
13	-37.1876	-12.5895	0.015	**0.014**	0.015	0.404	0.449	0.00019	0.070	0.074	**0.474**	2.466	**0.785**	-32.38
14	-37.6317	-12.8318	0.020	**0.020**	0.020	0.465	0.525	0.00036	0.054	0.061	**0.519**	2.581	**0.822**	-34.25
15	-37.6473	-12.9927	0.016	**0.016**	0.016	0.473	0.494	0.00004	0.024	0.025	**0.498**	2.564	**0.816**	-38.71
16	-37.7073	-12.8670	0.021	**0.021**	0.021	0.530	0.556	0.00012	0.047	0.047	**0.578**	2.731	**0.869**	-41.31
17	-37.3528	-12.3812	0.016	**0.016**	0.016	0.385	0.428	0.00024	0.053	0.056	**0.439**	2.361	**0.752**	-17.35
18	-37.3474	-12.3915	0.032	**0.032**	0.032	0.541	0.614	0.00028	0.077	0.081	**0.619**	2.854	**0.908**	-23.18
19	-37.4790	-12.2744	0.009	**0.009**	0.009	0.374	0.393	0.00007	0.034	0.035	**0.409**	2.287	**0.728**	-25.90
20	-37.5445	-13.1358	0.019	**0.017**	0.018	0.409	0.439	0.00092	0.095	0.099	**0.504**	2.544	**0.810**	-36.49
21	-37.5508	-13.0955	0.015	**0.015**	0.015	0.523	0.550	0.00004	0.029	0.030	**0.553**	2.678	**0.852**	-37.54
22	-37.5417	-13.0859	0.010	**0.010**	0.010	0.440	0.454	0.00004	0.023	0.023	**0.464**	2.437	**0.776**	-36.76
23	-37.4886	-13.0611	0.014	**0.013**	0.014	0.471	0.496	0.00018	0.043	0.045	**0.515**	2.633	**0.838**	-33.88
24	-37.4748	-13.1594	0.022	**0.022**	0.022	0.663	0.697	0.00019	0.042	0.044	**0.706**	3.048	**0.970**	-35.78
25	-37.2732	-12.5295	0.036	**0.036**	0.036	0.562	0.654	0.00014	0.027	0.030	**0.590**	2.904	**0.924**	-21.81
26	-37.3365	-12.0158	0.027	**0.027**	0.027	0.545	0.607	0.00014	0.068	0.069	**0.614**	2.812	**0.895**	-38.46
27	-37.3667	-12.9914	0.010	**0.010**	0.010	0.639	0.645	0.00002	0.032	0.032	**0.672**	2.964	**0.943**	-36.00
28	-36.5445	-11.0435	1.777	**1.770**	1.773	9.182	10.209	0.00324	0.458	0.492	**9.676**	12.24	**3.896**	-28.64

Supplementary Table 1 continued..

#	Lat [DDD]	Lon [DDD]	Total volume [km³]	**Net volume [km³]**	Cut volume [km³]	Cut area [km²]	Cut area 3D [km²]	Fill volume [km³]	Fill area [km²]	Fill area 3D [km²]	**Enclosed area [km²]**	Peri-meter [km]	**Mean diameter [km]**	Back-scatter [dB]
29	-36.9710	-13.1186	0.334	**0.334**	0.334	3.370	4.453	0.00010	0.060	0.062	**3.883**	7.71	**2.454**	-21.72
30	-36.9896	-13.1330	0.099	**0.098**	0.098	1.209	1.357	0.00010	0.039	0.042	**1.249**	4.195	**1.335**	-29.68
31	-36.9880	-13.2420	0.185	**0.185**	0.185	2.183	2.402	0.00021	0.083	0.085	**2.269**	6.121	**1.948**	-22.24
32	-36.9730	-12.4083	0.443	**0.442**	0.442	3.737	4.241	0.00056	0.188	0.194	**3.932**	8.405	**2.675**	-31.32
33	-37.0031	-13.2489	0.063	**0.062**	0.063	1.672	1.716	0.00049	0.152	0.155	**1.826**	4.948	**1.575**	-28.62
34	-37.0048	-13.1731	0.099	**0.098**	0.099	1.279	1.439	0.00066	0.151	0.160	**1.432**	4.434	**1.411**	-32.37
35	-36.9891	-12.3900	0.197	**0.197**	0.197	2.200	2.423	0.00011	0.065	0.067	**2.269**	5.938	**1.890**	-28.03
36	-37.0199	-13.2199	0.089	**0.089**	0.089	1.013	1.167	0.00019	0.036	0.041	**1.050**	3.847	**1.225**	-29.24
37	-37.0095	-12.4033	0.053	**0.053**	0.053	0.790	0.892	0.00011	0.042	0.044	**0.834**	3.278	**1.043**	-28.62
38	-37.0517	-13.1637	0.110	**0.108**	0.109	1.582	1.741	0.00108	0.150	0.162	**1.734**	5.277	**1.680**	-27.21
39	-37.0362	-12.4235	0.317	**0.316**	0.316	2.163	2.605	0.00043	0.121	0.126	**2.289**	5.748	**1.830**	-31.66
40	-37.0991	-13.0731	0.128	**0.127**	0.127	1.347	1.571	0.00050	0.099	0.105	**1.449**	4.545	**1.447**	-35.09
41	-37.1071	-12.4678	0.120	**0.118**	0.119	1.891	1.996	0.00085	0.245	0.252	**2.140**	5.467	**1.740**	-21.21
42	-37.1390	-13.0074	0.668	**0.667**	0.668	4.588	5.140	0.00072	0.171	0.179	**4.766**	8.797	**2.800**	-25.45
43	-37.1472	-12.7829	0.667	**0.666**	0.666	4.785	5.185	0.00041	0.135	0.140	**4.927**	8.491	**2.703**	-26.30
44	-37.1502	-12.4933	0.213	**0.211**	0.212	2.428	2.579	0.00090	0.173	0.179	**2.606**	5.979	**1.903**	-27.98
45	-37.1766	-13.3865	0.054	**0.053**	0.054	0.846	0.942	0.00050	0.105	0.109	**0.952**	3.574	**1.138**	-35.29
46	-37.1786	-12.9108	0.034	**0.034**	0.034	0.640	0.700	0.00010	0.022	0.023	**0.663**	2.9	**0.923**	-32.80
47	-37.1857	-13.4235	0.121	**0.121**	0.121	1.780	1.892	0.00005	0.042	0.043	**1.824**	4.989	**1.588**	-34.86
48	-37.1946	-13.3709	0.582	**0.581**	0.581	4.848	5.222	0.00050	0.187	0.192	**5.042**	8.626	**2.746**	-29.32
49	-37.2220	-12.4216	0.013	**0.011**	0.012	0.532	0.545	0.00107	0.122	0.131	**0.655**	2.944	**0.937**	-29.74
50	-37.2504	-12.3942	0.317	**0.317**	0.317	3.282	3.705	0.00017	0.096	0.100	**3.385**	9.065	**2.885**	-28.57
51	-37.2818	-12.2438	0.047	**0.047**	0.047	1.032	1.095	0.00011	0.051	0.052	**1.085**	3.891	**1.239**	-28.70
52	-37.2970	-12.3411	0.186	**0.185**	0.185	1.755	2.021	0.00023	0.057	0.060	**1.815**	5.185	**1.650**	-24.92
53	-37.2998	-12.1765	0.318	**0.318**	0.318	3.210	3.472	0.00024	0.098	0.101	**3.315**	6.758	**2.151**	-31.06
54	-37.3052	-12.0701	0.294	**0.293**	0.293	3.167	3.459	0.00040	0.142	0.146	**3.317**	7.167	**2.281**	-29.86
55	-37.3517	-13.2270	0.143	**0.143**	0.143	1.793	1.949	0.00030	0.098	0.101	**1.894**	4.968	**1.581**	-26.43
56	-37.3498	-12.1991	0.072	**0.071**	0.072	2.184	2.218	0.00016	0.074	0.075	**2.263**	5.854	**1.863**	-32.60

Supplementary Table 1 continued..

#	Lat [DDD]	Lon [DDD]	Total volume [km³]	**Net volume [km³]**	Cut volume [km³]	Cut area [km²]	Cut area 3D [km²]	Fill volume [km³]	Fill area [km²]	Fill area 3D [km²]	**Enclosed area [km²]**	Peri-meter [km]	**Mean diameter [km]**	Back-scatter [dB]
57	-37.3680	-12.1575	0.043	**0.043**	0.043	0.849	0.906	0.00010	0.035	0.036	**0.886**	3.429	**1.091**	-30.05
58	-37.3969	-13.2295	0.057	**0.056**	0.056	1.068	1.150	0.00039	0.097	0.099	**1.167**	3.934	**1.252**	-29.78
59	-37.3776	-12.0790	0.238	**0.237**	0.237	2.425	2.699	0.00052	0.111	0.119	**2.542**	6.321	**2.012**	-30.35
60	-37.3824	-12.0379	0.199	**0.198**	0.198	1.683	2.005	0.00054	0.095	0.108	**1.782**	4.867	**1.549**	-26.04
61	-37.3840	-12.1187	0.355	**0.355**	0.355	3.566	3.835	0.00020	0.111	0.112	**3.685**	7.243	**2.306**	-35.68
62	-37.4007	-12.1651	0.524	**0.522**	0.523	4.580	5.008	0.00082	0.196	0.203	**4.786**	8.482	**2.700**	-30.63
63	-37.4354	-13.2500	0.662	**0.661**	0.661	5.289	5.805	0.00062	0.179	0.184	**5.476**	8.782	**2.795**	-33.32
64	-37.4462	-12.6636	0.509	**0.508**	0.509	3.982	4.438	0.00050	0.151	0.158	**4.141**	8.204	**2.611**	-28.00
65	-37.4576	-12.9381	0.163	**0.162**	0.162	1.934	2.112	0.00075	0.098	0.105	**2.035**	5.452	**1.735**	-32.52
66	-37.4602	-12.9163	0.153	**0.151**	0.152	1.797	5.169	0.00082	0.115	0.299	**2.052**	5.425	**1.727**	-23.62
67	-37.5326	-12.8931	0.171	**0.166**	0.169	2.598	2.739	0.00221	0.223	0.239	**2.826**	6.639	**2.113**	-34.93
68	-37.6133	-12.5588	0.424	**0.423**	0.423	3.331	3.714	0.00055	0.171	0.179	**3.509**	7.445	**2.370**	-29.83
69	-37.7509	-13.6063	0.130	**0.115**	0.123	1.877	2.024	0.00768	0.489	0.526	**2.369**	5.601	**1.783**	-34.67
70	-37.7501	-13.5965	0.079	**0.076**	0.078	1.428	1.562	0.00164	0.181	0.197	**1.610**	5.766	**1.835**	-35.37
71	-38.0884	-14.4732	0.654	**0.647**	0.650	5.402	5.765	0.00362	0.247	0.284	**5.655**	8.513	**2.710**	-20.17
72	-38.3217	-14.5792	1.225	**1.207**	1.216	6.685	7.622	0.00907	0.539	0.599	**7.231**	9.692	**3.085**	-27.36
73	-38.2029	-14.7699	0.451	**0.451**	0.451	3.077	3.539	0.00026	0.066	0.070	**3.146**	6.391	**2.034**	-24.88
74	-36.8623	-13.1645	0.207	**0.206**	0.207	2.760	2.961	0.00044	0.109	0.113	**2.872**	6.047	**1.925**	-37.30
75	-36.8446	-13.1705	0.021	**0.020**	0.021	0.645	0.669	0.00063	0.117	0.120	**0.763**	3.112	**0.991**	-37.24
76	-36.8742	-13.2169	0.071	**0.070**	0.071	1.369	1.443	0.00030	0.070	0.072	**1.440**	4.422	**1.408**	-32.38
77	-36.9235	-13.1462	0.085	**0.084**	0.084	1.661	1.737	0.00022	0.061	0.064	**1.725**	5.142	**1.637**	-36.49
78	-36.9123	-13.1767	0.060	**0.060**	0.060	1.576	1.611	0.00002	0.030	0.030	**1.608**	4.638	**1.476**	-37.02
79	-37.0261	-13.3037	0.260	**0.252**	0.256	2.715	3.856	0.00382	0.314	0.333	**3.052**	6.261	**1.993**	-22.63
80	-37.0069	-13.2869	0.243	**0.243**	0.243	2.181	2.435	0.00017	0.044	0.047	**2.227**	5.362	**1.707**	-24.25
81	-37.0169	-13.1983	0.425	**0.417**	0.421	3.235	3.640	0.00402	0.343	0.361	**3.582**	6.773	**2.156**	-25.86
82	-37.0230	-13.1824	0.154	**0.150**	0.152	1.947	2.172	0.00219	0.271	0.291	**2.221**	5.398	**1.718**	-26.90
83	-37.1498	-12.9664	0.406	**0.397**	0.401	3.094	3.483	0.00452	0.361	0.379	**3.460**	6.677	**2.125**	-29.08
84	-37.1515	-12.9504	0.147	**0.140**	0.144	1.580	1.755	0.00331	0.319	0.339	**1.902**	5.16	**1.642**	-31.37

Supplementary Table 1 continued..

#	Lat [DDD]	Lon [DDD]	Total volume [km³]	**Net volume [km³]**	Cut volume [km³]	Cut area [km²]	Cut area 3D [km²]	Fill volume [km³]	Fill area [km²]	Fill area 3D [km²]	**Enclosed area [km²]**	Peri-meter [km]	**Mean diameter [km]**	Back-scatter [dB]
85	-37.1488	-12.9352	0.023	**0.023**	0.023	0.548	0.588	0.00015	0.066	0.067	**0.615**	2.805	**0.893**	-35.22
86	-37.2117	-12.8050	1.493	**1.492**	1.492	6.989	8.113	0.00035	0.109	0.116	**7.110**	9.597	**3.055**	-23.49
87	-37.0983	-12.6937	0.384	**0.382**	0.383	3.250	3.696	0.00136	0.281	0.293	**3.538**	6.778	**2.158**	-27.32
88	-37.1113	-12.6909	0.147	**0.141**	0.144	1.992	2.174	0.00342	0.451	0.478	**2.448**	5.813	**1.850**	-30.00
89	-36.9972	-12.7307	0.170	**0.169**	0.169	4.642	4.678	0.00066	0.167	0.169	**4.817**	7.87	**2.505**	-34.65
90	-37.0109	-12.7466	0.135	**0.134**	0.134	2.917	2.992	0.00028	0.094	0.097	**3.015**	6.231	**1.983**	-31.64
91	-36.9217	-12.4223	0.363	**0.362**	0.362	3.857	4.079	0.00070	0.213	0.219	**4.077**	7.803	**2.484**	-29.61
92	-37.0762	-12.4330	0.285	**0.271**	0.278	3.103	3.420	0.00675	0.451	0.481	**3.561**	6.796	**2.163**	-26.19
93	-37.0614	-12.4356	0.154	**0.151**	0.152	2.565	2.826	0.00145	0.162	0.179	**2.732**	6.167	**1.963**	-23.53
94	-36.8996	-12.2314	0.026	**0.026**	0.026	0.646	0.690	0.00024	0.091	0.092	**0.738**	3.059	**0.974**	-28.18
95	-36.9409	-12.2646	0.376	**0.375**	0.376	3.696	3.988	0.00011	0.074	0.078	**3.778**	7.403	**2.356**	-29.75
96	-36.9630	-12.2704	0.062	**0.061**	0.061	0.889	1.009	0.00038	0.072	0.074	**0.962**	3.544	**1.128**	-24.36
97	-36.9253	-12.3307	0.051	**0.051**	0.051	0.924	1.029	0.00030	0.117	0.122	**1.044**	3.654	**1.163**	-35.74
98	-37.0902	-12.8370	0.774	**0.772**	0.773	5.338	6.028	0.00105	0.164	0.171	**5.512**	9.133	**2.907**	-28.07
99	-37.1157	-12.8707	0.386	**0.381**	0.383	3.287	3.638	0.00258	0.269	0.290	**3.562**	7.176	**2.284**	-28.67
100	-37.0713	-12.8997	0.217	**0.215**	0.216	2.132	2.428	0.00064	0.096	0.103	**2.232**	5.726	**1.823**	-26.94
101	-37.0988	-12.9362	0.114	**0.111**	0.112	1.959	2.061	0.00137	0.185	0.191	**2.148**	5.236	**1.667**	-32.59
102	-37.1057	-12.9260	0.079	**0.076**	0.077	1.577	1.655	0.00174	0.277	0.286	**1.857**	4.964	**1.580**	-33.24
103	-37.1054	-12.9493	0.160	**0.159**	0.159	1.866	2.055	0.00080	0.153	0.159	**2.022**	5.222	**1.662**	-29.00
104	-37.1235	-12.9830	0.391	**0.387**	0.389	3.528	3.959	0.00195	0.314	0.337	**3.847**	7.064	**2.249**	-25.56
105	-37.1263	-12.9704	0.049	**0.045**	0.047	0.866	0.957	0.00168	0.182	0.202	**1.049**	3.693	**1.176**	-32.74
106	-37.1545	-13.0561	0.414	**0.412**	0.413	2.820	3.195	0.00099	0.118	0.124	**2.943**	6.182	**1.968**	-29.01
107	-37.1579	-13.0946	0.478	**0.477**	0.477	3.477	3.890	0.00041	0.083	0.089	**3.565**	6.946	**2.211**	-31.10
108	-37.1946	-13.1063	0.072	**0.071**	0.071	1.124	1.242	0.00019	0.052	0.056	**1.178**	3.89	**1.238**	-36.15
109	-37.1910	-13.1181	0.056	**0.055**	0.056	0.793	0.919	0.00059	0.102	0.113	**0.896**	3.402	**1.083**	-37.70
110	-37.1695	-13.1475	0.082	**0.082**	0.082	1.112	1.253	0.00039	0.062	0.068	**1.176**	3.927	**1.250**	-34.31
111	-37.1781	-13.1538	0.076	**0.076**	0.076	0.966	1.093	0.00038	0.085	0.088	**1.052**	3.674	**1.169**	-32.04
112	-37.2085	-13.2437	0.123	**0.122**	0.122	1.392	1.542	0.00018	0.067	0.069	**1.461**	4.404	**1.402**	-32.75

Supplementary Table 1 continued..

#	Lat [DDD]	Lon [DDD]	Total volume [km³]	**Net volume [km³]**	Cut volume [km³]	Cut area [km²]	Cut area 3D [km²]	Fill volume [km³]	Fill area [km²]	Fill area 3D [km²]	**Enclosed area [km²]**	Peri-meter [km]	**Mean diameter [km]**	Back-scatter [dB]
113	-37.2139	-13.2287	0.139	**0.138**	0.138	1.467	1.669	0.00052	0.101	0.106	**1.570**	4.489	**1.429**	-36.84
114	-37.2140	-13.2764	0.091	**0.090**	0.090	1.340	1.445	0.00050	0.112	0.115	**1.454**	4.33	**1.378**	-32.96
115	-37.2156	-13.2899	0.033	**0.033**	0.033	0.904	0.930	0.00025	0.069	0.070	**0.975**	3.509	**1.117**	-30.80
116	-37.2059	-13.3313	0.131	**0.130**	0.130	1.584	1.729	0.00035	0.074	0.077	**1.659**	4.609	**1.467**	-27.23
117	-37.1752	-13.3312	0.318	**0.316**	0.317	3.021	3.310	0.00099	0.133	0.148	**3.158**	6.681	**2.127**	-31.99
118	-37.2587	-13.3604	0.214	**0.210**	0.212	2.502	2.677	0.00180	0.338	0.342	**2.843**	6.008	**1.912**	-29.63
119	-37.2628	-13.3783	0.134	**0.133**	0.133	1.473	1.647	0.00048	0.096	0.099	**1.571**	4.48	**1.426**	-26.08
120	-37.2935	-13.4094	0.034	**0.034**	0.034	0.681	0.750	0.00025	0.094	0.096	**0.776**	3.135	**0.998**	-24.87
121	-37.2808	-13.4180	0.154	**0.152**	0.153	1.939	2.130	0.00075	0.158	0.163	**2.100**	5.429	**1.728**	-25.25
122	-37.3492	-13.6522	0.369	**0.366**	0.367	2.783	3.103	0.00113	0.184	0.190	**2.970**	6.15	**1.958**	-29.21
123	-37.3536	-13.6286	0.252	**0.249**	0.250	2.485	2.759	0.00164	0.155	0.165	**2.644**	6.215	**1.978**	-23.52
124	-37.5321	-13.7224	0.216	**0.213**	0.215	2.006	2.282	0.00136	0.274	0.280	**2.283**	5.374	**1.711**	-33.50
125	-37.5365	-13.5957	0.390	**0.390**	0.390	3.349	3.689	0.00025	0.086	0.088	**3.439**	6.709	**2.136**	-27.05
126	-37.5256	-13.4913	0.091	**0.090**	0.090	2.196	2.257	0.00071	0.255	0.257	**2.454**	5.764	**1.835**	-35.26
127	-37.4207	-13.3632	0.988	**0.984**	0.986	6.184	6.816	0.00217	0.290	0.300	**6.483**	9.332	**2.970**	-26.17
128	-37.3504	-13.1978	0.025	**0.024**	0.024	0.587	0.634	0.00030	0.100	0.101	**0.688**	2.953	**0.940**	-30.46
129	-37.3314	-13.2513	0.144	**0.137**	0.140	1.585	1.777	0.00326	0.295	0.311	**1.882**	4.898	**1.559**	-21.74
130	-37.3268	-13.2673	0.165	**0.163**	0.164	1.778	1.954	0.00092	0.111	0.116	**1.892**	5.044	**1.606**	-21.69
131	-37.3256	-13.2814	0.036	**0.016**	0.026	0.611	0.679	0.01009	0.510	0.546	**1.122**	3.779	**1.203**	-25.74
132	-37.3271	-13.2918	0.184	**0.170**	0.177	1.601	1.793	0.00690	0.308	0.332	**1.912**	4.981	**1.586**	-24.94
133	-37.2987	-13.2892	0.293	**0.291**	0.292	2.433	2.744	0.00074	0.133	0.138	**2.569**	5.702	**1.815**	-25.65
134	-37.2784	-13.2503	0.081	**0.078**	0.080	1.301	1.393	0.00140	0.212	0.215	**1.514**	4.565	**1.453**	-26.03
135	-37.3115	-13.3436	0.047	**0.034**	0.040	1.132	1.178	0.00648	0.409	0.424	**1.543**	4.463	**1.421**	-20.26
136	-37.3184	-13.3420	0.027	**0.024**	0.025	0.637	0.673	0.00179	0.249	0.255	**0.887**	3.36	**1.070**	-23.42
137	-37.3526	-13.4017	0.039	**0.039**	0.039	0.938	1.282	0.00003	0.010	0.011	**0.953**	3.489	**1.111**	-39.99
138	-37.1309	-13.2662	0.396	**0.394**	0.395	2.825	3.234	0.00102	0.096	0.104	**2.925**	6.213	**1.978**	-29.17
139	-37.1589	-13.2341	0.095	**0.094**	0.095	1.181	1.339	0.00067	0.109	0.111	**1.291**	4.065	**1.294**	-24.72
140	-37.2290	-13.1395	0.773	**0.763**	0.768	4.738	5.225	0.00521	0.388	0.402	**5.133**	8.15	**2.594**	-26.57

Supplementary Table 1 continued..

#	Lat [DDD]	Lon [DDD]	Total volume [km³]	**Net volume [km³]**	Cut volume [km³]	Cut area [km²]	Cut area 3D [km²]	Fill volume [km³]	Fill area [km²]	Fill area 3D [km²]	**Enclosed area [km²]**	Peri-meter [km]	**Mean diameter [km]**	Back-scatter [dB]
141	-37.2299	-13.1594	0.075	**0.073**	0.074	0.950	1.067	0.00088	0.142	0.149	**1.094**	3.745	**1.192**	-25.05
142	-37.2213	-13.1554	0.077	**0.077**	0.077	1.023	1.154	0.00040	0.079	0.086	**1.103**	3.873	**1.233**	-24.72
143	-37.2119	-13.0797	0.160	**0.159**	0.159	1.830	2.030	0.00038	0.075	0.078	**1.908**	4.938	**1.572**	-26.53
144	-37.2677	-13.1475	0.525	**0.523**	0.524	3.949	4.395	0.00082	0.223	0.230	**4.178**	7.391	**2.353**	-30.54
145	-37.2774	-13.1613	0.101	**0.099**	0.100	1.331	1.468	0.00104	0.137	0.146	**1.469**	4.334	**1.380**	-26.86
146	-37.3993	-12.9380	0.198	**0.196**	0.197	2.486	2.649	0.00120	0.219	0.229	**2.710**	5.911	**1.882**	-32.20
147	-37.4245	-13.0178	0.113	**0.112**	0.112	1.317	1.479	0.00059	0.093	0.100	**1.413**	4.321	**1.375**	-30.34
148	-37.3531	-12.9946	0.096	**0.095**	0.095	1.429	1.535	0.00008	0.049	0.050	**1.480**	4.326	**1.377**	-35.95
149	-37.3646	-12.9738	0.205	**0.203**	0.204	2.850	3.160	0.00062	0.168	0.173	**3.022**	7.184	**2.287**	-30.59
150	-37.6088	-12.8333	0.562	**0.558**	0.560	4.170	4.585	0.00202	0.269	0.282	**4.447**	7.502	**2.388**	-28.15
151	-37.6154	-12.7942	0.279	**0.278**	0.279	2.158	2.485	0.00059	0.117	0.123	**2.278**	5.398	**1.718**	-22.94
152	-37.6147	-12.9351	0.714	**0.713**	0.713	4.850	5.373	0.00068	0.168	0.175	**5.026**	8.012	**2.550**	-31.07
153	-37.6340	-12.8063	0.559	**0.554**	0.557	3.448	3.886	0.00215	0.240	0.255	**3.694**	6.977	**2.221**	-24.25
154	-37.7252	-12.6207	0.040	**0.040**	0.040	0.709	0.786	0.00042	0.124	0.125	**0.835**	3.244	**1.033**	-34.80
155	-37.7338	-12.6163	0.078	**0.076**	0.077	1.127	1.253	0.00117	0.230	0.233	**1.359**	4.176	**1.329**	-33.55
156	-37.7702	-12.5252	0.197	**0.191**	0.194	2.438	2.666	0.00301	0.346	0.360	**2.790**	6.327	**2.014**	-39.47
157	-38.4995	-11.4926	0.900	**0.890**	0.895	7.271	8.170	0.00518	0.775	0.804	**8.073**	12.662	**4.030**	-32.27
158	-36.9191	-12.3159	0.349	**0.349**	0.349	3.602	3.926	0.00013	0.065	0.068	**3.674**	7.417	**2.361**	-32.51
159	-36.9621	-12.1426	0.040	**0.040**	0.040	0.776	0.832	0.00020	0.060	0.060	**0.838**	3.311	**1.054**	-32.83
160	-36.9928	-12.1698	0.043	**0.043**	0.043	0.643	0.758	0.00022	0.074	0.077	**0.719**	3.013	**0.959**	-30.16
161	-37.1056	-12.4541	0.040	**0.038**	0.039	1.105	1.131	0.00075	0.169	0.171	**1.276**	4.044	**1.287**	-20.04
162	-37.1804	-12.4870	0.064	**0.063**	0.063	1.302	1.366	0.00018	0.089	0.091	**1.394**	4.524	**1.440**	-27.19
163	-37.1930	-12.4367	0.023	**0.023**	0.023	0.505	0.557	0.00007	0.015	0.017	**0.521**	2.567	**0.817**	-23.69
164	-37.2526	-12.5004	0.196	**0.195**	0.195	1.593	1.825	0.00050	0.107	0.116	**1.703**	4.652	**1.481**	-25.38
165	-37.2709	-12.5081	0.182	**0.182**	0.182	1.917	2.208	0.00021	0.062	0.068	**1.983**	5.023	**1.599**	-23.13
166	-37.2950	-12.4308	0.058	**0.050**	0.054	0.874	0.985	0.00398	0.437	0.457	**1.313**	4.075	**1.297**	-23.49
167	-37.2744	-12.4169	0.045	**0.042**	0.043	0.701	0.809	0.00154	0.189	0.204	**0.892**	3.378	**1.075**	-25.38
168	-37.3069	-12.4116	0.054	**0.054**	0.054	0.746	0.827	0.00009	0.040	0.042	**0.788**	3.156	**1.005**	-21.74

Supplementary Table 1 continued..

#	Lat [DDD]	Lon [DDD]	Total volume [km³]	**Net volume [km³]**	Cut volume [km³]	Cut area [km²]	Cut area 3D [km²]	Fill volume [km³]	Fill area [km²]	Fill area 3D [km²]	**Enclosed area [km²]**	Peri-meter [km]	**Mean diameter [km]**	Back-scatter [dB]
169	-37.3160	-12.4264	0.044	**0.041**	0.042	0.773	0.888	0.00158	0.275	0.293	**1.051**	3.641	**1.159**	-26.45
170	-37.1268	-12.7557	0.216	**0.216**	0.216	2.784	2.944	0.00007	0.051	0.051	**2.840**	6.32	**2.012**	-32.00
171	-37.1226	-12.7903	0.060	**0.060**	0.060	1.282	1.332	0.00007	0.031	0.032	**1.315**	4.166	**1.326**	-37.88
172	-37.1878	-12.7427	0.404	**0.404**	0.404	3.001	3.452	0.00029	0.073	0.079	**3.079**	6.323	**2.013**	-18.61
173	-37.1949	-12.6549	0.222	**0.213**	0.218	1.976	2.293	0.00463	0.263	0.289	**2.242**	5.379	**1.712**	-25.05
174	-37.0353	-12.9243	0.171	**0.165**	0.168	1.793	2.016	0.00287	0.250	0.262	**2.047**	5.103	**1.624**	-37.26
175	-37.0473	-12.9677	0.363	**0.353**	0.358	2.969	3.401	0.00531	0.386	0.414	**3.360**	6.553	**2.086**	-33.43
176	-37.0282	-12.9635	0.434	**0.417**	0.425	3.814	4.311	0.00877	0.488	0.533	**4.308**	8.043	**2.560**	-27.73
177	-37.0371	-12.9645	0.028	**-0.018**	0.005	0.283	0.306	0.02331	0.687	0.752	**0.972**	3.522	**1.121**	-31.06
178	-37.0552	-12.9958	0.386	**0.384**	0.385	3.146	3.571	0.00054	0.115	0.122	**3.266**	6.614	**2.105**	-30.26
179	-37.0160	-12.9679	0.155	**0.154**	0.154	2.179	2.433	0.00060	0.131	0.139	**2.314**	6.009	**1.913**	-30.20
180	-37.0194	-13.0261	0.262	**0.258**	0.260	2.601	2.904	0.00233	0.207	0.225	**2.812**	6.196	**1.972**	-27.52
181	-37.0358	-13.0270	0.347	**0.345**	0.346	2.978	3.278	0.00087	0.101	0.110	**3.084**	6.337	**2.017**	-27.65
182	-37.0240	-13.1216	0.703	**0.689**	0.696	4.984	5.668	0.00686	0.479	0.526	**5.469**	8.869	**2.823**	-29.76
183	-37.0167	-13.1045	0.501	**0.488**	0.495	3.284	3.712	0.00656	0.391	0.430	**3.680**	6.961	**2.216**	-25.82
184	-37.0284	-13.2428	0.034	**0.031**	0.032	0.664	0.722	0.00139	0.172	0.178	**0.837**	3.279	**1.044**	-27.25
185	-37.0749	-13.2442	0.167	**0.166**	0.166	1.762	1.970	0.00059	0.137	0.142	**1.901**	5.24	**1.668**	-31.97
186	-37.0968	-13.1641	0.229	**0.228**	0.228	2.547	2.776	0.00041	0.092	0.096	**2.642**	6.28	**1.999**	-24.04
187	-37.0775	-13.0646	0.053	**0.050**	0.052	0.818	0.930	0.00120	0.127	0.137	**0.947**	3.483	**1.109**	-29.75
188	-37.0865	-13.0347	0.517	**0.512**	0.514	3.934	4.534	0.00294	0.274	0.293	**4.214**	7.605	**2.421**	-29.12
189	-37.0845	-12.9993	0.444	**0.438**	0.441	3.880	4.438	0.00295	0.345	0.368	**4.231**	7.657	**2.437**	-31.52
190	-37.1160	-13.0402	0.056	**0.055**	0.055	0.934	1.020	0.00077	0.192	0.195	**1.127**	3.845	**1.224**	-30.67
191	-37.1194	-13.0261	0.082	**0.078**	0.080	1.181	1.301	0.00233	0.185	0.194	**1.367**	4.309	**1.372**	-28.98
192	-37.2532	-12.9490	0.098	**0.096**	0.097	1.250	1.610	0.00090	0.108	0.128	**1.361**	4.225	**1.345**	-33.39
193	-37.2410	-12.9351	0.232	**0.232**	0.232	2.706	2.945	0.00019	0.082	0.085	**2.792**	6.04	**1.923**	-32.42
194	-37.2807	-12.9736	0.535	**0.534**	0.534	4.107	4.656	0.00083	0.185	0.197	**4.299**	8.428	**2.683**	-24.00
195	-37.3010	-12.9516	0.073	**0.072**	0.072	0.991	1.145	0.00051	0.086	0.092	**1.078**	3.877	**1.234**	-24.05
196	-37.3092	-12.9894	0.866	**0.865**	0.865	6.826	7.255	0.00051	0.161	0.168	**6.997**	9.596	**3.055**	-30.59

Supplementary Table 1 continued..

#	Lat [DDD]	Lon [DDD]	Total volume [km³]	**Net volume [km³]**	Cut volume [km³]	Cut area [km²]	Cut area 3D [km²]	Fill volume [km³]	Fill area [km²]	Fill area 3D [km²]	**Enclosed area [km²]**	Peri-meter [km]	**Mean diameter [km]**	Back-scatter [dB]
197	-37.3121	-13.0334	0.516	**0.513**	0.515	3.562	3.955	0.00155	0.198	0.214	**3.765**	6.953	**2.213**	-22.32
198	-37.3408	-13.1774	0.016	**0.016**	0.016	0.558	0.583	0.00015	0.074	0.075	**0.633**	2.828	**0.900**	-30.78
199	-37.5791	-13.2596	0.319	**0.318**	0.318	2.730	2.986	0.00046	0.072	0.077	**2.806**	5.953	**1.895**	-23.81
200	-37.6400	-13.2457	0.562	**0.556**	0.559	4.169	4.605	0.00280	0.280	0.299	**4.455**	7.624	**2.427**	-20.79
201	-37.6597	-13.2574	0.373	**0.365**	0.369	4.217	4.458	0.00429	0.397	0.415	**4.621**	7.739	**2.463**	-19.13
202	-37.6321	-13.1661	0.385	**0.378**	0.382	3.926	4.178	0.00358	0.367	0.381	**4.300**	7.741	**2.464**	-25.73
203	-37.8707	-13.7061	0.223	**0.222**	0.222	1.819	2.108	0.00052	0.054	0.062	**1.875**	4.885	**1.555**	-35.64
204	-37.8744	-13.6908	0.059	**0.054**	0.057	1.170	1.236	0.00236	0.202	0.213	**1.374**	4.289	**1.365**	-32.20
205	-37.8930	-13.6973	0.438	**0.435**	0.437	4.059	4.428	0.00142	0.170	0.178	**4.233**	8.172	**2.601**	-29.56
206	-37.9087	-13.7616	0.033	**0.032**	0.032	0.614	0.676	0.00019	0.077	0.078	**0.692**	2.975	**0.947**	-38.79
207	-37.5616	-13.7945	0.060	**0.057**	0.059	1.016	1.128	0.00152	0.188	0.197	**1.205**	3.909	**1.244**	-33.15
208	-37.5536	-13.7557	0.115	**0.112**	0.114	1.608	1.793	0.00106	0.275	0.280	**1.885**	4.922	**1.567**	-33.48
209	-37.6672	-13.6280	0.343	**0.337**	0.340	2.790	3.103	0.00292	0.374	0.397	**3.167**	6.431	**2.047**	-28.10
210	-37.7413	-13.6547	0.048	**0.046**	0.047	0.908	0.994	0.00105	0.171	0.178	**1.080**	3.701	**1.178**	-32.97
211	-37.7405	-13.7192	0.412	**0.408**	0.410	3.790	4.136	0.00204	0.311	0.320	**4.105**	7.45	**2.371**	-31.30
212	-37.7476	-13.6899	0.368	**0.363**	0.365	4.277	4.571	0.00241	0.366	0.388	**4.648**	7.906	**2.517**	-34.68
213	-37.7921	-13.6312	0.053	**0.050**	0.051	1.841	1.869	0.00123	0.354	0.358	**2.197**	5.282	**1.681**	-40.45
214	-37.8049	-13.6826	0.689	**0.688**	0.689	4.806	5.430	0.00030	0.081	0.089	**4.893**	8.732	**2.779**	-29.83
215	-37.8365	-13.5341	0.086	**0.086**	0.086	1.630	1.719	0.00023	0.110	0.112	**1.743**	4.844	**1.542**	-31.56
216	-37.8531	-13.6530	0.189	**0.187**	0.188	2.479	2.721	0.00104	0.195	0.203	**2.677**	6.352	**2.022**	-26.81
217	-37.9747	-14.3093	0.071	**0.070**	0.070	1.716	1.791	0.00045	0.138	0.142	**1.856**	4.846	**1.543**	-27.29
218	-37.5695	-12.0778	0.163	**0.162**	0.162	1.679	1.887	0.00030	0.077	0.078	**1.759**	4.744	**1.510**	-29.86
219	-37.5613	-12.0956	0.100	**0.100**	0.100	1.323	1.517	0.00021	0.108	0.110	**1.435**	4.277	**1.361**	-38.38
220	-37.4970	-12.0299	0.411	**0.408**	0.409	3.270	3.672	0.00150	0.233	0.247	**3.512**	6.691	**2.130**	-27.79
221	-37.4801	-12.0330	0.063	**0.062**	0.063	1.014	1.129	0.00042	0.110	0.115	**1.127**	3.804	**1.211**	-26.52
222	-37.4919	-12.0679	0.344	**0.343**	0.344	2.627	3.013	0.00086	0.126	0.138	**2.760**	5.989	**1.906**	-22.90
223	-37.4912	-12.0512	0.052	**0.048**	0.050	1.155	1.248	0.00226	0.279	0.293	**1.437**	4.28	**1.362**	-25.53
224	-37.5097	-12.0182	0.134	**0.126**	0.130	1.370	1.641	0.00404	0.355	0.386	**1.729**	4.712	**1.500**	-33.40

Supplementary Table 1 continued..

#	Lat [DDD]	Lon [DDD]	Total volume [km³]	**Net volume [km³]**	Cut volume [km³]	Cut area [km²]	Cut area 3D [km²]	Fill volume [km³]	Fill area [km²]	Fill area 3D [km²]	**Enclosed area [km²]**	Peri-meter [km]	**Mean diameter [km]**	Back-scatter [dB]
225	-37.5326	-12.0180	0.855	**0.832**	0.843	5.202	5.951	0.01147	0.462	0.504	**5.678**	8.687	**2.765**	-36.81
226	-37.5176	-12.0318	0.130	**0.118**	0.124	1.555	1.726	0.00615	0.383	0.408	**1.942**	5.001	**1.592**	-27.96
227	-37.5334	-11.9877	0.058	**0.057**	0.057	1.028	1.088	0.00015	0.056	0.058	**1.086**	3.707	**1.180**	-38.87
228	-37.5277	-11.9795	0.025	**0.024**	0.025	0.689	0.725	0.00036	0.108	0.110	**0.799**	3.187	**1.014**	-37.99
229	-37.5626	-11.9708	0.029	**0.028**	0.029	0.889	0.916	0.00008	0.072	0.073	**0.963**	3.566	**1.135**	-38.67
230	-37.5406	-11.9964	0.037	**0.037**	0.037	0.987	1.025	0.00010	0.037	0.038	**1.026**	3.623	**1.153**	-39.19
231	-37.5758	-12.1038	0.049	**0.048**	0.049	1.166	1.249	0.00014	0.101	0.102	**1.270**	4.011	**1.277**	-37.36
234	-37.5199	-12.0870	0.063	**0.054**	0.059	0.905	1.019	0.00425	0.260	0.283	**1.168**	3.852	**1.226**	-31.39
235	-37.5119	-12.0605	1.024	**1.021**	1.022	7.496	8.231	0.00174	0.262	0.283	**7.777**	10.973	**3.493**	-23.93
236	-37.5517	-12.0496	0.094	**0.093**	0.093	1.849	1.953	0.00074	0.160	0.164	**2.013**	5.084	**1.618**	-40.09
237	-37.5403	-12.0411	0.097	**0.095**	0.096	1.594	1.712	0.00109	0.263	0.269	**1.861**	5.033	**1.602**	-31.60
238	-37.5352	-12.0615	0.164	**0.155**	0.159	2.038	2.234	0.00427	0.321	0.346	**2.365**	5.591	**1.780**	-31.99
239	-37.5463	-12.1180	0.407	**0.405**	0.406	3.634	4.033	0.00065	0.129	0.135	**3.772**	7.209	**2.295**	-28.65
240	-37.5527	-12.1378	0.053	**0.052**	0.052	0.951	1.037	0.00059	0.101	0.105	**1.054**	3.7	**1.178**	-24.87
241	-37.6014	-11.9461	0.058	**0.058**	0.058	1.102	1.154	0.00006	0.028	0.029	**1.133**	3.792	**1.207**	-34.81
242	-37.5992	-12.1040	0.025	**0.024**	0.024	0.720	0.796	0.00014	0.120	0.121	**0.842**	3.272	**1.042**	-39.12
243	-37.6058	-11.9960	0.050	**0.045**	0.048	0.915	0.999	0.00246	0.306	0.314	**1.224**	4.001	**1.274**	-38.78
244	-37.5795	-12.0718	0.031	**0.031**	0.031	0.817	0.849	0.00008	0.037	0.037	**0.855**	3.318	**1.056**	-39.53
245	-37.4915	-12.1303	0.035	**0.034**	0.035	0.799	0.850	0.00011	0.060	0.061	**0.861**	3.31	**1.054**	-33.10
246	-37.5221	-11.9874	0.050	**0.048**	0.049	0.925	0.977	0.00088	0.091	0.099	**1.019**	3.64	**1.159**	-38.77
247	-37.5155	-12.1736	0.156	**0.152**	0.154	3.706	3.776	0.00209	0.340	0.349	**4.055**	7.262	**2.312**	-28.73
248	-37.5502	-12.3572	0.059	**0.059**	0.059	1.002	1.062	0.00008	0.027	0.029	**1.031**	3.61	**1.149**	-22.44
249	-37.5374	-12.3037	0.276	**0.271**	0.274	2.731	2.974	0.00219	0.271	0.283	**3.008**	6.353	**2.022**	-31.67
250	-37.4811	-12.4669	0.193	**0.193**	0.193	1.860	2.174	0.00024	0.055	0.060	**1.919**	4.936	**1.571**	-24.99
251	-37.5669	-12.5444	0.170	**0.169**	0.170	1.997	2.201	0.00062	0.197	0.201	**2.199**	5.461	**1.738**	-21.07
252	-37.5836	-12.4061	0.265	**0.264**	0.264	2.419	2.709	0.00069	0.122	0.131	**2.546**	5.776	**1.839**	-29.16
253	-37.6090	-12.5305	0.181	**0.177**	0.179	2.017	2.271	0.00225	0.168	0.182	**2.189**	5.469	**1.741**	-29.03
254	-37.5995	-12.5285	0.037	**0.029**	0.033	0.733	0.788	0.00440	0.305	0.320	**1.040**	3.71	**1.181**	-26.33

Supplementary Table 1 continued..

#	Lat [DDD]	Lon [DDD]	Total volume [km³]	**Net volume [km³]**	Cut volume [km³]	Cut area [km²]	Cut area 3D [km²]	Fill volume [km³]	Fill area [km²]	Fill area 3D [km²]	**Enclosed area [km²]**	Peri-meter [km]	**Mean diameter [km]**	Back-scatter [dB]
255	-37.5832	-12.5375	0.174	**0.170**	0.172	2.318	2.517	0.00158	0.202	0.211	**2.525**	6.042	**1.923**	-28.53
256	-37.5198	-12.4326	0.067	**0.067**	0.067	1.044	1.152	0.00026	0.065	0.068	**1.111**	3.752	**1.194**	-19.44
257	-37.4881	-12.3367	0.128	**0.128**	0.128	1.961	2.153	0.00002	0.022	0.023	**1.986**	5.884	**1.873**	-27.18
258	-37.5026	-12.3773	0.204	**0.204**	0.204	2.447	2.704	0.00025	0.070	0.075	**2.523**	6.553	**2.086**	-21.71
259	-37.5093	-12.3915	0.194	**0.191**	0.193	1.799	2.039	0.00173	0.143	0.151	**1.946**	5.021	**1.598**	-21.84
260	-37.4853	-12.3992	0.125	**0.125**	0.125	2.012	2.134	0.00013	0.062	0.066	**2.079**	5.512	**1.755**	-22.89
261	-37.4913	-12.4200	0.052	**0.052**	0.052	0.895	1.011	0.00001	0.013	0.014	**0.910**	3.72	**1.184**	-22.54
262	-37.435	-12.337	39.018	**38.930**	38.974	90.253	98.238	0.04362	2.672	2.859	**93.125**	40.719	**12.961**	-27.10
263	-37.4320	-12.2712	0.088	**0.088**	0.088	1.048	1.197	0.00009	0.018	0.019	**1.068**	3.836	**1.221**	-24.30
264	-37.4160	-12.2697	0.233	**0.232**	0.232	2.990	3.230	0.00060	0.109	0.117	**3.107**	6.418	**2.043**	-29.55
265	-37.3755	-12.5205	0.481	**0.480**	0.481	3.114	3.586	0.00034	0.088	0.095	**3.208**	6.479	**2.062**	NoData
266	-37.3953	-12.5311	0.234	**0.234**	0.234	2.144	2.454	0.00016	0.071	0.076	**2.219**	5.371	**1.710**	NoData
267	-37.4148	-12.5412	0.283	**0.276**	0.280	2.208	2.592	0.00356	0.256	0.283	**2.469**	5.729	**1.824**	NoData
268	-37.4376	-12.5516	0.123	**0.121**	0.122	1.193	1.431	0.00098	0.093	0.101	**1.288**	4.048	**1.289**	NoData
269	-37.4367	-12.5391	0.046	**0.045**	0.046	0.729	0.816	0.00048	0.064	0.068	**0.795**	3.219	**1.025**	NoData
270	-37.4499	-12.5415	0.074	**0.069**	0.072	0.720	0.915	0.00235	0.149	0.184	**0.870**	3.349	**1.066**	-37.54
271	-37.3476	-12.5141	0.021	**0.020**	0.021	0.542	0.595	0.00022	0.098	0.102	**0.642**	2.862	**0.911**	NoData
272	-37.5134	-12.5220	0.177	**0.176**	0.176	1.885	2.136	0.00044	0.073	0.077	**1.962**	5.438	**1.731**	-23.47
273	-37.5165	-12.4815	0.188	**0.188**	0.188	1.945	2.140	0.00007	0.048	0.049	**1.997**	5.303	**1.688**	-23.19
274	-37.5199	-12.5465	0.053	**0.052**	0.052	1.022	1.102	0.00015	0.056	0.058	**1.081**	3.853	**1.226**	-26.00
275	-37.4912	-12.5358	0.063	**0.063**	0.063	1.015	1.127	0.00004	0.039	0.040	**1.056**	3.792	**1.207**	-21.19
276	-37.4903	-12.4906	0.022	**0.022**	0.022	0.480	0.532	0.00006	0.025	0.027	**0.506**	2.555	**0.813**	-24.98
277	-37.4741	-12.6057	0.152	**0.152**	0.152	1.740	1.982	0.00019	0.075	0.079	**1.818**	5.169	**1.645**	-19.25
278	-37.4910	-12.5538	0.040	**0.040**	0.040	0.939	0.998	0.00017	0.077	0.080	**1.018**	3.657	**1.164**	-25.26
279	-37.565	-12.743	69.756	**69.704**	69.730	138.840	171.590	0.02620	2.051	2.385	**145.770**	55.303	**17.603**	-24.62
280	-37.5044	-12.6976	0.111	**0.110**	0.111	1.263	1.426	0.00025	0.044	0.049	**1.309**	4.373	**1.392**	-24.14
281	-37.4681	-12.7510	0.104	**0.103**	0.104	1.351	1.518	0.00028	0.043	0.047	**1.396**	4.556	**1.450**	-24.67
282	-37.4906	-12.8432	0.241	**0.240**	0.241	2.759	3.007	0.00050	0.159	0.162	**2.923**	6.245	**1.988**	-36.60

Supplementary Table 1 continued..

#	Lat [DDD]	Lon [DDD]	Total volume [km³]	**Net volume [km³]**	Cut volume [km³]	Cut area [km²]	Cut area 3D [km²]	Fill volume [km³]	Fill area [km²]	Fill area 3D [km²]	**Enclosed area [km²]**	Peri-meter [km]	**Mean diameter [km]**	Back-scatter [dB]
283	-37.4746	-12.8192	0.038	**0.038**	0.038	0.607	0.686	0.00004	0.022	0.022	**0.630**	2.847	**0.906**	-26.44
284	-37.5440	-12.8151	0.031	**0.031**	0.031	0.530	0.606	0.00013	0.037	0.041	**0.567**	2.702	**0.860**	-23.70
285	-37.6121	-12.6845	0.205	**0.200**	0.203	2.041	2.358	0.00271	0.256	0.282	**2.301**	6.123	**1.949**	-25.96
286	-37.6204	-12.6789	0.115	**0.111**	0.113	1.711	1.969	0.00211	0.225	0.250	**1.939**	5.783	**1.841**	-26.09
287	-37.6470	-12.6977	1.059	**1.058**	1.058	6.084	6.892	0.00036	0.072	0.082	**6.167**	9.368	**2.982**	-27.02
288	-37.6464	-12.6275	0.028	**0.027**	0.027	0.651	0.703	0.00063	0.074	0.083	**0.726**	3.05	**0.971**	-34.59
289	-37.5936	-12.8795	0.049	**0.049**	0.049	1.285	1.321	0.00004	0.040	0.040	**1.327**	4.117	**1.310**	-41.91
290	-37.6334	-12.8623	0.026	**0.026**	0.026	0.601	0.637	0.00005	0.019	0.020	**0.622**	2.81	**0.894**	-35.48
291	-37.5915	-12.9391	0.209	**0.206**	0.208	4.120	4.269	0.00130	0.222	0.233	**4.348**	7.681	**2.445**	-39.82
292	-37.6587	-12.9734	0.028	**0.028**	0.028	0.845	0.870	0.00003	0.027	0.027	**0.873**	3.37	**1.073**	-38.54
293	-37.6743	-12.9742	0.019	**0.019**	0.019	0.743	0.763	0.00018	0.096	0.097	**0.840**	3.289	**1.047**	-36.71
294	-37.7207	-12.9238	0.109	**0.109**	0.109	1.963	2.033	0.00007	0.044	0.045	**2.010**	5.478	**1.744**	-38.39
295	-37.7368	-12.9497	0.032	**0.031**	0.032	0.695	0.739	0.00004	0.025	0.025	**0.721**	3.064	**0.975**	-40.92
296	-37.7260	-12.9730	0.158	**0.157**	0.157	2.219	2.385	0.00018	0.071	0.074	**2.294**	5.808	**1.849**	-33.57
297	-37.7198	-12.8623	0.022	**0.022**	0.022	0.718	0.750	0.00022	0.044	0.046	**0.764**	3.342	**1.064**	-38.68
298	-37.7492	-12.8463	0.073	**0.072**	0.073	1.443	1.521	0.00085	0.105	0.111	**1.550**	4.46	**1.420**	-32.23
299	-37.6566	-12.5547	0.047	**0.047**	0.047	1.276	1.359	0.00039	0.141	0.143	**1.420**	5.511	**1.754**	-34.82
300	-37.4261	-12.7206	0.095	**0.094**	0.095	1.093	1.227	0.00074	0.099	0.108	**1.194**	3.955	**1.259**	-27.48
301	-37.3225	-13.0545	0.083	**0.079**	0.081	1.318	1.461	0.00213	0.300	0.314	**1.620**	4.895	**1.558**	-25.10
302	-37.3548	-13.0737	0.025	**0.025**	0.025	0.578	0.623	0.00012	0.043	0.044	**0.622**	2.848	**0.907**	-33.80
303	-37.3184	-13.1375	0.072	**0.072**	0.072	1.447	1.543	0.00002	0.028	0.029	**1.477**	4.993	**1.589**	-27.54
304	-37.3297	-13.1062	0.075	**0.074**	0.074	1.446	1.567	0.00076	0.149	0.154	**1.597**	5.613	**1.787**	-25.61
305	-37.5201	-13.6046	0.344	**0.333**	0.339	3.460	3.726	0.00577	0.343	0.373	**3.808**	7.008	**2.231**	-27.06
306	-37.5492	-13.7755	0.038	**0.038**	0.038	0.920	0.964	0.00008	0.031	0.031	**0.952**	3.5	**1.114**	-33.83
307	-37.6461	-13.9358	1.712	**1.697**	1.704	12.101	12.833	0.00747	0.673	0.719	**12.787**	12.933	**4.117**	-32.48
308	-37.7098	-13.9631	0.151	**0.150**	0.151	1.814	1.947	0.00058	0.105	0.109	**1.920**	4.945	**1.574**	-24.39
309	-37.8536	-14.0271	1.197	**1.188**	1.192	7.216	7.933	0.00430	0.341	0.375	**7.565**	10.539	**3.355**	-26.47
310	-37.9823	-14.0310	0.056	**0.056**	0.056	1.311	1.353	0.00005	0.023	0.024	**1.335**	4.119	**1.311**	-35.65

Supplementary Table 1 continued..

#	Lat [DDD]	Lon [DDD]	Total volume [km³]	**Net volume [km³]**	Cut volume [km³]	Cut area [km²]	Cut area 3D [km²]	Fill volume [km³]	Fill area [km²]	Fill area 3D [km²]	**Enclosed area [km²]**	Peri-meter [km]	**Mean diameter [km]**	Back-scatter [dB]
311	-37.9686	-14.0426	0.067	**0.065**	0.066	1.281	1.352	0.00085	0.083	0.092	**1.366**	4.526	**1.441**	-34.08
312	-38.0372	-14.0849	0.135	**0.135**	0.135	2.901	4.178	0.00008	0.038	0.069	**3.241**	6.556	**2.087**	-31.95
313	-38.0049	-13.9204	0.051	**0.050**	0.050	1.411	1.440	0.00034	0.095	0.096	**1.507**	4.414	**1.405**	-31.38
314	-38.3037	-14.6635	0.049	**0.049**	0.049	0.944	1.020	0.00019	0.074	0.076	**1.019**	3.634	**1.157**	-24.61
315	-38.3193	-14.6414	0.094	**0.093**	0.093	2.041	2.101	0.00030	0.095	0.097	**2.138**	5.35	**1.703**	-24.64
316	-38.2827	-14.6381	0.322	**0.321**	0.321	2.737	3.087	0.00053	0.117	0.121	**2.857**	6.239	**1.986**	-22.42
317	-38.2678	-14.6503	0.152	**0.148**	0.150	2.776	2.904	0.00198	0.231	0.241	**3.009**	6.37	**2.028**	-23.70
318	-38.1139	-14.5970	0.182	**0.179**	0.181	2.522	2.672	0.00118	0.185	0.190	**2.709**	6.056	**1.928**	-24.48
319	-38.0894	-14.5257	0.401	**0.397**	0.399	3.650	3.955	0.00223	0.220	0.233	**3.874**	7.296	**2.322**	-27.78
320	-38.2061	-14.7966	0.246	**0.240**	0.243	4.896	5.090	0.00276	0.475	0.491	**5.376**	8.607	**2.740**	-27.75
321	-38.5057	-14.3878	1.998	**1.989**	1.994	11.393	12.645	0.00407	0.290	0.350	**11.695**	13.735	**4.372**	-34.54
322	-38.4877	-14.4357	0.597	**0.594**	0.595	5.243	5.867	0.00163	0.256	0.276	**5.504**	9.306	**2.962**	-32.13
323	-38.6129	-14.2380	0.069	**0.069**	0.069	1.029	1.161	0.00043	0.128	0.132	**1.159**	3.989	**1.270**	-36.52
324	-38.8037	-13.3989	0.410	**0.410**	0.410	3.414	3.821	0.00029	0.102	0.105	**3.522**	7.014	**2.233**	-36.23
325	-38.8994	-13.4129	0.097	**0.096**	0.097	1.432	1.545	0.00047	0.061	0.068	**1.496**	4.515	**1.437**	-28.43
326	-38.8511	-13.3748	0.053	**0.053**	0.053	0.807	0.898	0.00009	0.041	0.042	**0.849**	3.291	**1.048**	-34.65
327	-38.7436	-13.2283	0.384	**0.383**	0.383	3.306	3.620	0.00070	0.183	0.186	**3.495**	6.827	**2.173**	-34.17
328	-38.7376	-13.1883	0.199	**0.198**	0.199	2.731	2.930	0.00068	0.108	0.114	**2.843**	6.353	**2.022**	-32.93
329	-38.5875	-13.1875	0.031	**0.029**	0.030	0.562	0.664	0.00067	0.078	0.089	**0.641**	2.858	**0.910**	-40.20
330	-38.4491	-11.3081	0.999	**0.994**	0.996	7.601	8.429	0.00234	0.292	0.315	**7.922**	11.15	**3.549**	-30.64
331	-37.5264	-9.7283	15.539	**15.504**	15.521	66.044	71.695	0.01727	1.025	1.095	**67.482**	40.086	**12.760**	-25.64
332	-37.1302	-10.0937	2.212	**2.207**	2.210	14.593	15.663	0.00258	0.332	0.365	**15.005**	16.006	**5.095**	-25.25
333	-35.5822	-11.4615	0.337	**0.337**	0.337	2.345	2.729	0.00005	0.021	0.023	**2.372**	5.593	**1.780**	-34.07
334	-35.5472	-11.5123	2.397	**2.394**	2.396	17.438	18.275	0.00137	0.239	0.264	**17.727**	16.437	**5.232**	-29.79
335	-35.5861	-11.5045	1.382	**1.367**	1.374	13.812	14.569	0.00786	0.408	0.521	**14.261**	16.348	**5.204**	-36.30
336	-36.9807	-12.2671	0.052	**0.052**	0.052	0.800	0.906	0.00000	0.004	0.004	**0.805**	3.434	**1.093**	-23.37
337	-36.9976	-12.2715	0.099	**0.099**	0.099	1.342	1.520	0.00006	0.030	0.032	**1.376**	4.507	**1.435**	-23.42
338	-37.0088	-12.2523	0.029	**0.029**	0.029	0.666	0.753	0.00028	0.082	0.087	**0.750**	3.282	**1.045**	-25.75

Supplementary Table 1 continued..

#	Lat [DDD]	Lon [DDD]	Total volume [km³]	**Net volume [km³]**	Cut volume [km³]	Cut area [km²]	Cut area 3D [km²]	Fill volume [km³]	Fill area [km²]	Fill area 3D [km²]	**Enclosed area [km²]**	Peri-meter [km]	**Mean diameter [km]**	Back-scatter [dB]
339	-37.0254	-12.3239	0.305	**0.305**	0.305	3.087	3.498	0.00008	0.070	0.074	**3.164**	6.469	**2.059**	-23.43
340	-37.0381	-12.3552	0.125	**0.124**	0.125	1.498	1.716	0.00060	0.061	0.067	**1.563**	4.562	**1.452**	-22.88
341	-36.9844	-12.3380	0.093	**0.092**	0.093	1.982	2.103	0.00022	0.072	0.076	**2.058**	5.632	**1.793**	-26.76
342	-37.0105	-12.2764	0.025	**0.024**	0.025	0.641	0.706	0.00034	0.086	0.093	**0.728**	3.211	**1.022**	-26.28
343	-37.0863	-12.1423	0.149	**0.149**	0.149	1.963	2.216	0.00018	0.061	0.066	**2.029**	5.745	**1.829**	-23.27
344	-37.1314	-12.1464	0.503	**0.502**	0.503	3.746	4.235	0.00014	0.054	0.057	**3.809**	7.488	**2.384**	-22.80
345	-37.1069	-12.1265	0.586	**0.586**	0.586	5.308	5.741	0.00043	0.129	0.137	**5.450**	8.704	**2.771**	-23.13
346	-37.1139	-12.1637	0.126	**0.124**	0.125	2.027	2.258	0.00095	0.245	0.261	**2.277**	5.664	**1.803**	-21.82
347	-37.1201	-12.0521	0.129	**0.128**	0.129	2.054	2.217	0.00051	0.118	0.124	**2.176**	5.932	**1.888**	-34.95
348	-37.1649	-12.0673	0.079	**0.079**	0.079	1.393	1.469	0.00011	0.039	0.041	**1.435**	4.342	**1.382**	-35.24
349	-37.1581	-12.0406	0.020	**0.020**	0.020	0.661	0.685	0.00006	0.040	0.041	**0.703**	3.001	**0.955**	-34.52
350	-37.1720	-12.0261	0.036	**0.035**	0.035	0.698	0.769	0.00037	0.076	0.078	**0.775**	3.129	**0.996**	-33.94
351	-37.1647	-11.9520	0.010	**0.010**	0.010	0.531	0.539	0.00007	0.042	0.042	**0.574**	2.692	**0.857**	-36.88
352	-37.1303	-11.9531	0.049	**0.049**	0.049	1.148	1.185	0.00005	0.026	0.027	**1.177**	3.94	**1.254**	-36.01
353	-37.0707	-12.0770	0.058	**0.057**	0.058	0.963	1.066	0.00051	0.093	0.099	**1.059**	3.702	**1.178**	-25.42
354	-37.0830	-12.0736	0.471	**0.467**	0.469	3.901	4.248	0.00213	0.233	0.241	**4.144**	7.704	**2.452**	-26.91
355	-37.2397	-12.0044	0.255	**0.254**	0.255	4.084	4.171	0.00043	0.119	0.123	**4.213**	7.633	**2.430**	-39.25
356	-37.2296	-12.1102	0.164	**0.164**	0.164	2.342	2.453	0.00007	0.048	0.050	**2.396**	5.529	**1.760**	-27.40
357	-37.2575	-12.1276	0.079	**0.078**	0.079	2.064	2.133	0.00022	0.072	0.075	**2.141**	5.602	**1.783**	-32.03
358	-37.2383	-12.1342	0.171	**0.170**	0.171	3.288	3.440	0.00041	0.083	0.092	**3.379**	7.132	**2.270**	-30.28
359	-37.2340	-12.1505	0.027	**0.027**	0.027	0.876	0.906	0.00035	0.080	0.082	**0.959**	3.496	**1.113**	-30.69
360	-37.2564	-12.2461	0.161	**0.159**	0.160	2.631	2.750	0.00095	0.250	0.256	**2.888**	6.274	**1.997**	-26.58
361	-37.2908	-12.2975	0.329	**0.328**	0.329	3.066	3.411	0.00028	0.091	0.093	**3.163**	7.039	**2.241**	-26.96
362	-37.2757	-12.3449	0.536	**0.536**	0.536	4.223	4.635	0.00016	0.057	0.059	**4.289**	7.692	**2.448**	-24.73
363	-37.3425	-12.4177	0.083	**0.081**	0.082	0.984	1.100	0.00084	0.143	0.148	**1.129**	3.815	**1.214**	-22.45
364	-37.3215	-12.4364	0.237	**0.234**	0.236	1.907	2.184	0.00151	0.125	0.135	**2.036**	5.103	**1.624**	-24.48
365	-37.3872	-12.3851	0.268	**0.268**	0.268	2.887	3.125	0.00012	0.040	0.042	**2.933**	6.899	**2.196**	-28.39
366	-37.3419	-12.3471	0.044	**0.044**	0.044	0.665	0.745	0.00017	0.052	0.054	**0.719**	3.026	**0.963**	-25.34

Supplementary Table 1 continued..

#	Lat [DDD]	Lon [DDD]	Total volume [km³]	**Net volume [km³]**	Cut volume [km³]	Cut area [km²]	Cut area 3D [km²]	Fill volume [km³]	Fill area [km²]	Fill area 3D [km²]	**Enclosed area [km²]**	Peri-meter [km]	**Mean diameter [km]**	Back-scatter [dB]
367	-37.3369	-12.3695	0.044	**0.042**	0.043	0.725	0.797	0.00063	0.103	0.110	**0.830**	3.236	**1.030**	-19.61
368	-37.3125	-12.3538	0.069	**0.069**	0.069	0.810	0.952	0.00004	0.010	0.012	**0.822**	3.302	**1.051**	-27.56
369	-37.3219	-12.3574	0.169	**0.169**	0.169	1.485	1.706	0.00010	0.026	0.028	**1.514**	4.433	**1.411**	-24.20
370	-37.3270	-12.3215	0.087	**0.085**	0.086	1.101	1.229	0.00060	0.104	0.111	**1.207**	4.225	**1.345**	-22.38
371	-37.3159	-12.3085	0.145	**0.144**	0.144	1.646	1.848	0.00050	0.103	0.107	**1.753**	5.076	**1.616**	-22.33
372	-37.4072	-12.4232	0.047	**0.045**	0.046	0.629	0.735	0.00063	0.128	0.134	**0.759**	3.102	**0.987**	-23.88
373	-37.3786	-12.4690	0.144	**0.141**	0.142	2.184	2.369	0.00161	0.335	0.347	**2.524**	5.763	**1.834**	-16.06
374	-37.7940	-12.7069	0.186	**0.185**	0.186	3.249	3.365	0.00045	0.116	0.119	**3.371**	6.652	**2.117**	-29.65
375	-37.7714	-12.7838	1.841	**1.820**	1.830	9.337	10.756	0.01052	0.779	0.839	**10.134**	12.473	**3.970**	-22.80
376	-37.7475	-12.7684	0.612	**0.610**	0.611	4.843	5.516	0.00128	0.160	0.176	**5.013**	9.383	**2.987**	-24.21
377	-37.7662	-12.9755	0.097	**0.096**	0.097	3.062	3.115	0.00023	0.091	0.094	**3.158**	6.858	**2.183**	-39.00
378	-37.4315	-11.9400	0.599	**0.597**	0.598	5.195	5.591	0.00120	0.137	0.149	**5.346**	8.83	**2.811**	-34.68
379	-37.4501	-11.9460	0.255	**0.254**	0.254	2.842	3.042	0.00059	0.093	0.098	**2.942**	6.461	**2.057**	-33.27
380	-37.4482	-12.0768	0.070	**0.070**	0.070	1.061	1.182	0.00022	0.053	0.057	**1.117**	3.798	**1.209**	-26.77
381	-37.4540	-12.0531	0.980	**0.970**	0.975	5.840	6.616	0.00507	0.360	0.398	**6.216**	8.977	**2.857**	-25.98
382	-37.4981	-12.2625	0.127	**0.126**	0.126	3.104	3.219	0.00070	0.175	0.181	**3.287**	6.624	**2.108**	-28.25
383	-37.5363	-13.1333	0.061	**0.060**	0.061	1.087	1.166	0.00024	0.031	0.038	**1.120**	3.822	**1.217**	-39.75
384	-37.5439	-13.0770	0.018	**0.018**	0.018	0.542	0.575	0.00031	0.084	0.086	**0.627**	2.836	**0.903**	-31.91
385	-37.5940	-13.1952	0.051	**0.051**	0.051	0.995	1.051	0.00015	0.049	0.051	**1.046**	3.664	**1.166**	-35.92
386	-37.6226	-13.1114	0.075	**0.075**	0.075	2.531	2.599	0.00045	0.222	0.225	**2.758**	7.231	**2.302**	-37.92
387	-37.6359	-12.9259	0.033	**0.032**	0.032	0.730	0.790	0.00017	0.044	0.046	**0.775**	3.283	**1.045**	-32.13
388	-37.1697	-13.0489	0.616	**0.611**	0.614	5.330	5.993	0.00221	0.266	0.284	**5.604**	10.976	**3.494**	-32.95
389	-37.0476	-13.1175	0.311	**0.307**	0.309	2.761	3.168	0.00197	0.253	0.272	**3.018**	7.102	**2.261**	-25.81
390	-37.0308	-13.0788	0.322	**0.306**	0.314	3.782	4.145	0.00810	0.543	0.572	**4.331**	8.187	**2.606**	-24.52
391	-37.1181	-13.1135	0.142	**0.142**	0.142	1.524	1.686	0.00007	0.027	0.028	**1.901**	4.937	**1.571**	-28.22
392	-37.1051	-13.1128	0.170	**0.167**	0.169	2.185	2.438	0.00107	0.208	0.217	**2.397**	6.141	**1.955**	-27.71
393	-37.0629	-13.1458	0.199	**0.194**	0.197	2.301	2.598	0.00247	0.227	0.257	**2.532**	6.128	**1.951**	-30.12
394	-37.1599	-12.7293	0.488	**0.488**	0.488	3.706	4.213	0.00020	0.056	0.061	**3.768**	7.89	**2.511**	-23.09

Supplementary Table 1 continued..

#	Lat [DDD]	Lon [DDD]	Total volume [km³]	**Net volume [km³]**	Cut volume [km³]	Cut area [km²]	Cut area 3D [km²]	Fill volume [km³]	Fill area [km²]	Fill area 3D [km²]	**Enclosed area [km²]**	Peri-meter [km]	**Mean diameter [km]**	Back-scatter [dB]
395	-37.1508	-12.6397	0.421	**0.420**	0.421	4.167	4.753	0.00058	0.111	0.125	**4.285**	9.089	**2.893**	-27.94
396	-37.1845	-12.6433	0.417	**0.408**	0.412	3.727	4.254	0.00452	0.403	0.439	**4.138**	7.655	**2.437**	-25.67
397	-37.2045	-12.6980	0.538	**0.538**	0.538	3.632	4.154	0.00032	0.064	0.070	**3.702**	7.435	**2.367**	-22.63
398	-37.2727	-12.4336	0.043	**0.042**	0.043	0.828	0.895	0.00034	0.075	0.079	**0.905**	3.424	**1.090**	-28.76
399	-37.2620	-12.4440	0.093	**0.093**	0.093	1.160	1.297	0.00004	0.013	0.014	**1.176**	4.069	**1.295**	-22.75
400	-37.3027	-12.4604	0.067	**0.065**	0.066	0.943	1.075	0.00069	0.206	0.216	**1.152**	3.886	**1.237**	-26.06
401	-37.3111	-12.4552	0.068	**0.068**	0.068	1.184	1.277	0.00000	0.007	0.007	**1.193**	3.942	**1.255**	-29.01
402	-37.2958	-12.4740	0.334	**0.331**	0.333	3.613	4.047	0.00154	0.226	0.237	**3.846**	7.841	**2.496**	-21.48
403	-37.3120	-12.4049	0.094	**0.079**	0.087	1.274	1.429	0.00742	0.459	0.502	**1.736**	4.871	**1.550**	-21.34
404	-37.3018	-12.3874	0.051	**0.048**	0.050	1.013	1.109	0.00139	0.181	0.195	**1.197**	4.156	**1.323**	-24.34
405	-37.3607	-12.2917	0.126	**0.125**	0.125	1.387	1.560	0.00018	0.039	0.041	**1.429**	4.298	**1.368**	-22.42
406	-37.3161	-12.2221	0.075	**0.075**	0.075	1.281	1.406	0.00011	0.055	0.056	**1.339**	4.621	**1.471**	-27.88
407	-37.3150	-12.0317	0.038	**0.038**	0.038	0.744	0.804	0.00013	0.045	0.046	**0.791**	3.172	**1.010**	-33.85
408	-37.4234	-12.0512	0.394	**0.380**	0.387	4.356	4.620	0.00664	0.538	0.574	**4.906**	7.99	**2.543**	-32.42
409	-37.4062	-12.0097	0.624	**0.618**	0.621	4.959	5.448	0.00283	0.370	0.389	**5.342**	8.368	**2.664**	-34.46
410	-37.4370	-11.9992	0.040	**0.039**	0.040	0.672	0.755	0.00033	0.046	0.053	**0.720**	3.039	**0.967**	-30.91
411	-37.6992	-12.2630	0.041	**0.040**	0.040	0.926	0.953	0.00022	0.050	0.051	**0.978**	3.562	**1.134**	-34.90
412	-37.4188	-12.8577	0.242	**0.241**	0.241	3.601	3.718	0.00034	0.096	0.099	**3.702**	7.089	**2.256**	-33.07
413	-37.4158	-12.9974	0.162	**0.162**	0.162	2.143	2.355	0.00042	0.130	0.134	**2.277**	6.107	**1.944**	-29.24
414	-37.4137	-12.8851	0.034	**0.033**	0.034	0.781	0.831	0.00012	0.050	0.052	**0.832**	3.284	**1.045**	-34.49
415	-37.4401	-12.9062	0.083	**0.081**	0.082	1.459	1.600	0.00093	0.151	0.168	**1.613**	4.729	**1.505**	-29.79
416	-37.4432	-13.0464	0.434	**0.431**	0.432	3.640	4.064	0.00180	0.184	0.204	**3.829**	7.241	**2.305**	-26.62
417	-37.3020	-12.9332	0.070	**0.069**	0.069	1.058	1.172	0.00006	0.029	0.030	**1.089**	3.844	**1.224**	-24.09
418	-37.4382	-12.7506	0.034	**0.033**	0.033	0.600	0.680	0.00015	0.078	0.079	**0.678**	2.968	**0.945**	-26.90
419	-37.4265	-12.8198	0.041	**0.041**	0.041	0.655	0.728	0.00003	0.024	0.025	**0.681**	2.948	**0.938**	-23.13
420	-37.4433	-12.8053	0.025	**0.024**	0.024	0.615	0.666	0.00027	0.047	0.051	**0.663**	3.096	**0.985**	-26.06
421	-37.5036	-12.9415	0.030	**0.030**	0.030	0.876	0.900	0.00003	0.028	0.029	**0.906**	3.451	**1.098**	-39.61
422	-37.8040	-13.7410	0.024	**0.024**	0.024	0.992	1.005	0.00003	0.020	0.021	**1.013**	3.61	**1.149**	-40.62

Supplementary Table 1 continued..

#	Lat [DDD]	Lon [DDD]	Total volume [km³]	**Net volume [km³]**	Cut volume [km³]	Cut area [km²]	Cut area 3D [km²]	Fill volume [km³]	Fill area [km²]	Fill area 3D [km²]	**Enclosed area [km²]**	Peri-meter [km]	**Mean diameter [km]**	Back-scatter [dB]
423	-37.7808	-13.6533	0.147	**0.144**	0.145	2.693	2.817	0.00112	0.224	0.228	**2.921**	6.199	**1.973**	-29.84
424	-37.2316	-12.4114	0.050	**0.049**	0.050	0.782	0.864	0.00044	0.102	0.105	**0.885**	3.376	**1.075**	-28.48
425	-37.2386	-12.4324	0.053	**0.053**	0.053	1.025	1.103	0.00022	0.064	0.067	**1.092**	3.732	**1.188**	-24.79
426	-37.2440	-12.4169	0.232	**0.226**	0.229	4.116	4.197	0.00305	0.438	0.451	**4.563**	7.811	**2.486**	-27.81
427	-37.0490	-12.8302	0.094	**0.094**	0.094	1.613	1.693	0.00004	0.026	0.027	**1.642**	4.871	**1.550**	-28.07
428	-37.1898	-12.9897	0.207	**0.206**	0.206	1.997	2.203	0.00055	0.138	0.140	**2.138**	5.237	**1.667**	-33.34
429	-37.0059	-13.2165	0.264	**0.264**	0.264	2.478	2.831	0.00021	0.057	0.061	**2.538**	6.746	**2.147**	-31.59
430	-37.0389	-13.2739	0.307	**0.305**	0.306	2.586	2.893	0.00088	0.108	0.121	**2.698**	5.966	**1.899**	-27.80

Supplementary Table 2: Decision table summarizing the factors used in the structural Benthic Terrain Model classification (modified after Erdey-Heydorn[4]). The Bathymetric Position Index (BPI) values are given in grid units (standardised value of each data point was multiplied by 100) and represent ±1 standard deviation. Slope values are given in degrees. For a better visualization, some of the structural zones are grouped together in the final map.

Class	Zone	Lower limit Broad BPI	Upper limit Broad BPI	Lower limit Fine BPI	Upper limit Fine BPI	Lower limit Slope	Upper limit Slope
1	Flat Plains	-100	100	-100	100		3
2	Broad Slopes	-100	100	-100	100	3	25
3	Steep Slopes	-100	100	-100	100	25	
4	Broad Depression		-100	-100	100		3
5	Narrow Depression		-100		-100		3
6	Local Ridge, Boulders, Pinnacles in Depression		-100	100			
7	Crevices, NarrowGullies over elevated terrain	100			-100		
8	Flat Ridge Tops	100		-100	100		3
9	Broad Slopes on Elevated Terrain	100		-100	100	3	25
10	Steep Slopes on Elevated Terrain	100		-100	100	25	
11	Rock Outcrop High, Narrow Ridge	100		100			
12	Scarp, Cliff	-100	100		-100	3	
13	Local Ridge, Boulders, Pinnacles on Broad Flats	-100	100	100			3
14	Local Ridge, Boulders, Pinnacles on Slopes	-100	100	100		3	
15	Local Depression, Current Scours	-100	100		-100		
16	Broad Slopes in Broad Depressions		-100	-100	100	3	25
17	Steep Slopes in Broad Depressions		-100	-100	100	25	
18	Broad Slopes in Narrow Depressions		-100		-100	3	25
19	Steep Slopes in Narrow Depressions		-100		-100	25	

Supplementary Notes

Supplementary Note 1 | Combined backscatter mosaic. A combined backscatter mosaic is presented, retrieved from two different cruises, both acquired with MBES of R/V MARIA S. MERIAN. In order to estimate errors when combining these data sets, we considered the following issues and excluded the backscatter data recorded from the EM1002 around Nightingale Island, due to different transducer geometry and frequency.

Seafloor acoustic reflectivities were recorded from the same vessel but during different cruises within 15 months during which time the MBES EM120 system was updated to EM122. According to Kongsberg's service report, the update in September/October 2012 saw the addition of two new transmit arrays and an upgrade of the MRU Seapath 300 to 320. The arrays and the MRU where installed at the same positions as their predecessors. Kongsberg Support stated, as expected, that the updates could lead to slight improvements in radiometric resolution due to better shaping of the TX (transmit) pulse on EM122.

Processing corrections for the two systems EM120 and EM122 are based on algorithms developed at the end of the last century, as described in more detail in Hammerstad[6]. Schimel et al.[7] mention that the manufacturer ensures normalization of the signal level during acquisition, most likely to a level that enables comparability of results with those of other Kongsberg systems. Examination of areas of overlap between the two surveys enabled us to calculate an average offset of about -2 dB between the EM122 and the EM120 datasets (Supplementary Figure 6).

The original histogram of differences between backscatter values in the overlap area is plotted in the lower right corner of Supplementary Figure 6. It reveals two distributions, a Gaussian normal distribution around 0 dB and a distribution with a positive skew (large tail to the right). The skewed distribution results from the aforementioned -2 dB shift. The histogram in the upper-right corner of Supplementary Figure 6 shows the distribution of differences after correction for this offset. The remaining Gaussian normal distribution is a result of a lateral displacement between the cruises of about 1-1.5 times the beam footprint of the 2° x 2° transmit array. Since this displacement seems related to the acquisition geometry, we assume that it occurs due to the absence of a Doppler effect correction in the EM120 data. This affects the outer range of the swath most strongly.

Further acoustic reflectivity differences can be related to heading effects, spherical spreading, frequency-dependent attenuation and level variation with angle of incidence at the seafloor.

These issues are, for most purposes, well corrected for in the Kongsberg MBES backscatter by the manufacturer by applying a rather complex dynamic gain and corrections, albeit based on simple models described in the guidelines and recommendations of the GeoHab Backscatter Working Group[8,9]. Ongoing investigations[10-12] of some of the remaining errors will probably only be solved after calibration with ground truth or additional calibrated acoustic devices.

Wherever possible, we try to avoid using near-nadir information, which are most likely influenced by direct reflection and therefore oversaturated. This is made possible using a processing feature of QPS Fledermaus FMGT™.

We decided to use backscatter dB values instead of a commonly used grey scale range and a color-coded classification instead of an unclassified grey scale. We found the transformation of backscatter [dB] values from normalized and TX/RX gain corrected systems like EM120 and EM122 to a graphical greyscale range of 0-254 values to be insufficient for our purposes. Instead, we utilized ESRI's *ArcGIS*™ image classification tool for supervised classification based on natural breaks in the backscatter mosaic histogram to generate a classified mosaic image. The color-coded results emphasize the different backscatter dB-ranges regardless of area covered, enhancing their visibility to the human eye. Several other publications deal with seabed backscatter in a similar way, e.g. Eason et al.[13].

Finally, yet importantly, we are aware of a certain penetration of the 12 kHz signal of the EM120/122 MBES depending on grain size and water saturation of the topmost seafloor sediments. This topic is the focus of ongoing research at the Seafloor Mapping and Imaging Group and the Marine Technology – Environmental Research Group at MARUM/University of Bremen. Based on comparison with ground-truthed data sets from the North Pond, Lucky Strike, and Azores areas in the North Atlantic, where water depth and volcanic background are similar to the Tristan da Cunha region, we expect penetration by about 1 m within coarser sandy sediments and about 2 m or more into fine-grained and pelagic sediments, depending on water saturation. In comparison to this, bedrock or volcanic outcrop, boulders, pebbles, and rubble create a much different angular independence and support strong backscatter in every direction. They usually correlate nicely with higher roughness values as derived from bathymetry.

Based on this, those unusual areas of relatively high backscatter intensity but relatively inconspicuous roughness values from low frequency MBES like the 12 kHz EM120/EM122 are of particular interest. In areas like this, it seems to be possible to identify sediment-draped gas and hydrate pockets, mudflows and lava flows.

Supplementary References

1. Geissler, W. H., Wintersteller, P., Strack, A., Kammann, J., Jegen, M., Marcia, M., Schloemer, A. & Jokat, W. GIS based analysis, processed sub bottom profiler, and composite grid and backscatter mosaics around Tristan da Cunha. PANGAEA, https://doi.pangaea.de/10.1594/PANGAEA.906154 (unpublished dataset) (2019).
2. Jenness, J. S. Calculating landscape surface area from digital elevation models. *Wildlife Society Bulletin* **32(3)**, 829-839 (2004)
3. Ryan, W. B. F., Carbotte, S. M., Coplan, J. O., O'Hara, S., Melkonian, A., Arko, R., Weissel, R. A., Ferrini, V., Goodwillie, A., Nitsche, F., Bonczkowski, J. & Zemsky, R. Global Multi-Resolution Topography (GMRT) synthesis. *Geochemistry, Geophysics, Geosystems* **10(3)**, doi:10.1029/2008GC002332 (2009).
4. Smith, W. H. F. & Sandwell, D. T. Global Sea Floor Topography from Satellite Altimetry and Ship Depth Soundings. *Science* **277(5334)**, 1956-1962, doi: 10.1126/science.277.5334.1956 (1997).
5. Erdey-Heydorn, M. D. An ArcGIS Seabed Characterization Toolbox Developed for Investigating Benthic Habitats. *Marine Geodesy* **31(4)**, 318–358, doi: 10.1080/ 01490410802466819 (2008).
6. Hammerstad, E. EM technical note: Backscattering and seabed image reflectivity. Horten, Norway: Kongsberg Maritime AS (2000).
7. Schimel, A. C. G., Beaudoin, J., Gaillot, A., Keith, G., Le Bas, T., Parnum, I. & Schmidt, V. Processing backscatter data: from datagrams to Angular responses and mosaics. In: Lurton, X., Lamarche, G. (Eds.) Backscatter measurements by seafloor-mapping sonars. Guidelines and Recommendations. A collective report by members of the GeoHab Backscatter Working Group. Geohab Report. 150p. Chap.6 pp.133-164 (2015).
8. Lurton, X. & Lamarche, G. (Eds.) Backscatter measurements by seafloor-mapping sonars; Guidelines and Recommendations. A collective report by

members of the GeoHab Backscatter Working Group https://niwa.co.nz/static/BWSG_REPORT_MAY2015_web.pdf (2015).

9. Schimel, A.C.G.,-Beaudoin, J., Parnum, I.M., Le Bas T., Schmidt, V., Keith, G. & Ierodiaconou, D. Multibeam sonar backscatter data processing. *Marine Geophysical Researches* **39**, 121-137, doi: 10.1007/s11001-018-9341-z (2018).
10. Ladroit, Y., Lamarche, G. & Pallentin, A. Seafloor multibeam backscatter calibration experiment: comparing 45°-tilted 38-kHz split-beam echosounder and 30-kHz multibeam data. *Marine Geophysical Researches* **39**, 41-53, doi: 10.1007/s11001-017-9340-5 (2018).
11. Lamarche, G. & Lurton, X. Recommendations for improved and coherent acquisition and processing of backscatter data from seafloor-mapping sonars. *Marine Geophysical Researches* **39**, 5-22. doi: 10.1007/s11001-017-9315-6 (2018).
12. Malik, M., Schimel, A. C. G., Masetti, G., Roche, M., Le Deunf, J., Dolan, M. F., Beaudoin, J., Augustin, J.-M., Hamilton, T. & Parnum, I. Results from the First Phase of the Seafloor Backscatter Processing Software Inter-Comparison Project. *Geosciences* 9, 516 (2019).
13. Eason, D. E., Dunn, R. A., Canales, J. P. & Sohn, R. A. Segment-scale variations in seafloor volcanic and tectonic processes from multibeam sonar imaging, Mid-Atlantic Ridge Rainbow region (35°45′–36°35′N). *Geochemistry, Geophysics, Geosystems* **17**, 3560– 3579, doi: 10.1002/2016GC006433 (2016).
